# Mobile Elements Harboring Heavy Metal and Bacitracin Resistance Genes Are Common among Listeria monocytogenes Strains Persisting on Dairy Farms

**DOI:** 10.1128/mSphere.00383-21

**Published:** 2021-07-07

**Authors:** Hanna Castro, François P. Douillard, Hannu Korkeala, Miia Lindström

**Affiliations:** a Department of Food Hygiene and Environmental Health, Faculty of Veterinary Medicine, University of Helsinki, Helsinki, Finland; University of Kentucky

**Keywords:** agroecosystems, antimicrobial resistance, biocide resistance, comparative genomics, environmental microbiology, food safety, heavy metal resistance, mobile genetic elements, One Health, persistence

## Abstract

Listeria monocytogenes is a foodborne pathogen and a resilient environmental saprophyte. Dairy farms are a reservoir of L. monocytogenes, and strains can persist on farms for years. Here, we sequenced the genomes of 250 L. monocytogenes isolates to investigate the persistence and mobile genetic elements (MGEs) of *Listeria* strains inhabiting dairy farms. We performed a single-nucleotide polymorphism (SNP)-based phylogenomic analysis to identify 14 monophyletic clades of L. monocytogenes persistent on the farms for ≥6 months. We found that prophages and other mobile genetic elements were, on average, more numerous among isolates in persistent than nonpersistent clades, and we demonstrated that resistance genes against bacitracin, arsenic, and cadmium were significantly more prevalent among isolates in persistent than nonpersistent clades. We identified a diversity of mobile elements among the 250 farm isolates, including three novel plasmids, three novel transposons, and a novel prophage harboring cadmium resistance genes. Several of the mobile elements we identified in Listeria were identical to the mobile elements of enterococci, which is indicative of recent transfer between these genera. Through a genome-wide association study, we discovered that three putative defense systems against invading prophages and plasmids were negatively associated with persistence on farms. Our findings suggest that mobile elements support the persistence of L. monocytogenes on dairy farms and that L. monocytogenes inhabiting the agroecosystem is a potential reservoir of mobile elements that may spread to the food industry.

**IMPORTANCE** Animal-derived raw materials are an important source of L. monocytogenes in the food industry. Knowledge of the factors contributing to the pathogen’s transmission and persistence on farms is essential for designing effective strategies against the spread of the pathogen from farm to fork. An increasing body of evidence suggests that mobile genetic elements support the adaptation and persistence of L. monocytogenes in the food industry, as these elements contribute to the dissemination of genes encoding favorable phenotypes, such as resilience against biocides. Understanding of the role of farms as a potential reservoir of these elements is needed for managing the transmission of mobile elements across the food chain. Because L. monocytogenes coinhabits the farm ecosystem with a diversity of other bacterial species, it is important to assess the degree to which genetic elements are exchanged between *Listeria* and other species, as such exchanges may contribute to the rise of novel resistance phenotypes.

## INTRODUCTION

Listeria monocytogenes leads a double life. In one, it is a potentially lethal, zoonotic foodborne pathogen, and in the other, a ubiquitous environmental saprophyte (1). Agroecosystems provide a favorable habitat for L. monocytogenes, and the pathogen is especially prevalent on dairy farms (2, 3). L. monocytogenes strains can inhabit dairy farms for years and be widely distributed in the farm environment, leading to the frequent contamination of milk (4, 5). Raw milk and animals destined for slaughter are a major contamination source in the food industry (6–8). Knowledge of the pathogen’s ecology on farms is essential for controlling the spread of L. monocytogenes from farms to the food industry.

L. monocytogenes is extremely resilient and can tolerate various stresses used in the food industry to control the pathogen (9, 10). These phenotypic traits enable L. monocytogenes to survive in food processing environments for years, a phenomenon known as persistence (11–15). Mobile genetic elements (MGEs) are common among L. monocytogenes isolates from food processing environments (14–16) and may harbor genes mediating tolerance to heat shock (17), salt and acid stress (18, 19), and biocides (20, 21). These findings led us to the hypothesis that mobile genetic elements play a key role in the environmental adaptation and persistence of L. monocytogenes.

Although dairy farms are considered a reservoir of L. monocytogenes (2) and are known to harbor hypervirulent strains (22), the era of next-generation sequencing has witnessed very few efforts to illuminate the pathogen’s ecology in the farm environment. How L. monocytogenes adapts to life in the farm ecosystem, and to what extent the farm environment acts as a source of mobile genetic elements for L. monocytogenes strains persisting in food processing environments, are key issues to explore. Such insights would be instrumental in developing novel strategies to reduce contamination on farms and in the raw materials delivered to the food industry.

Here, we sequenced the genomes of 250 L. monocytogenes isolates obtained from three Finnish dairy farms during 2013 to 2016 (5) to investigate the persistence and mobile genetic elements of L. monocytogenes in the farm environment. We performed a single-nucleotide polymorphism (SNP)-based phylogenomic analysis to group the isolates into persistent and nonpersistent clades and identified plasmids and chromosomal mobile elements among the 250 genomes. We found that prophages and other mobile genetic elements were, on average, more abundant among isolates in persistent clades than among those in nonpersistent clades, and that a significantly higher portion of isolates in persistent clades harbored genes against bacitracin, arsenic, and cadmium, compared to those in nonpersistent clades. Finally, we explored genome-wide associations between clusters of orthologous genes and persistence. We found that defense systems against invading prophages and plasmids, including the CRISPR-*cas* IIA system (23) and the type II restriction modification system Lmo3J (24), were negatively associated with persistence on farms. Taken together, our findings suggest that prophages and mobile genetic elements confer an ecological advantage for persistence on farms and that L. monocytogenes inhabiting the farm environment constitutes a reservoir of diverse mobile genetic elements that may spread upstream in the food chain.

## RESULTS

### Persisting clades of L. monocytogenes were detected on all three farms.

**FIG 1:**
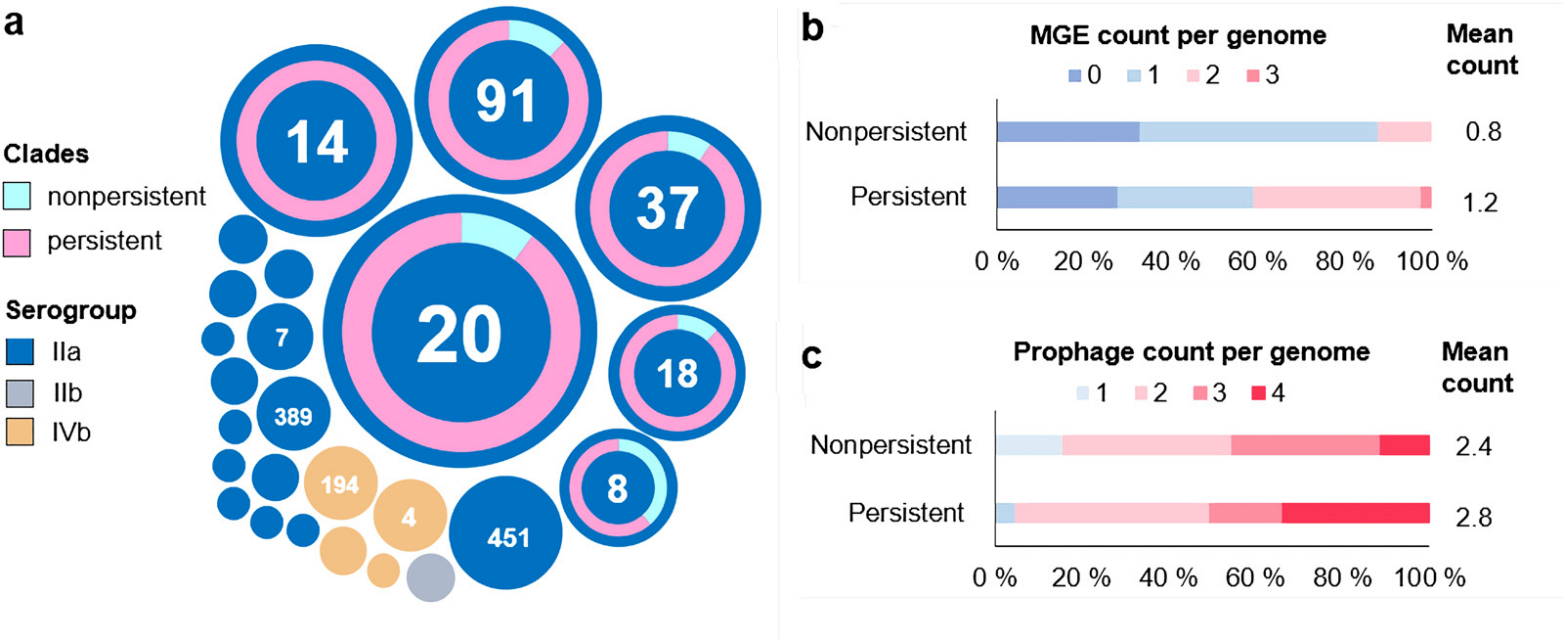
L. monocytogenes isolates in persistent clades contained, on average, more prophages and other mobile genetic elements (MGEs) than isolates in nonpersistent clades. (a) L. monocytogenes isolates in this study represented 25 unique sequence types (STs), and persistent clades were detected among the six most prevalent STs. Each circle represents a unique ST, and the area of the circle corresponds to the number of isolates. For each ST in which persistent clades were detected, doughnut charts illustrate the proportion of isolates in persistent (pink) and nonpersistent (aquamarine) clades. (b, c) Distribution of isolates by numbers of nonphage MGEs (b) and prophages (c) per genome among isolates in persistent and nonpersistent clades. The average number of the elements per genome is also given.

**FIG 2:**
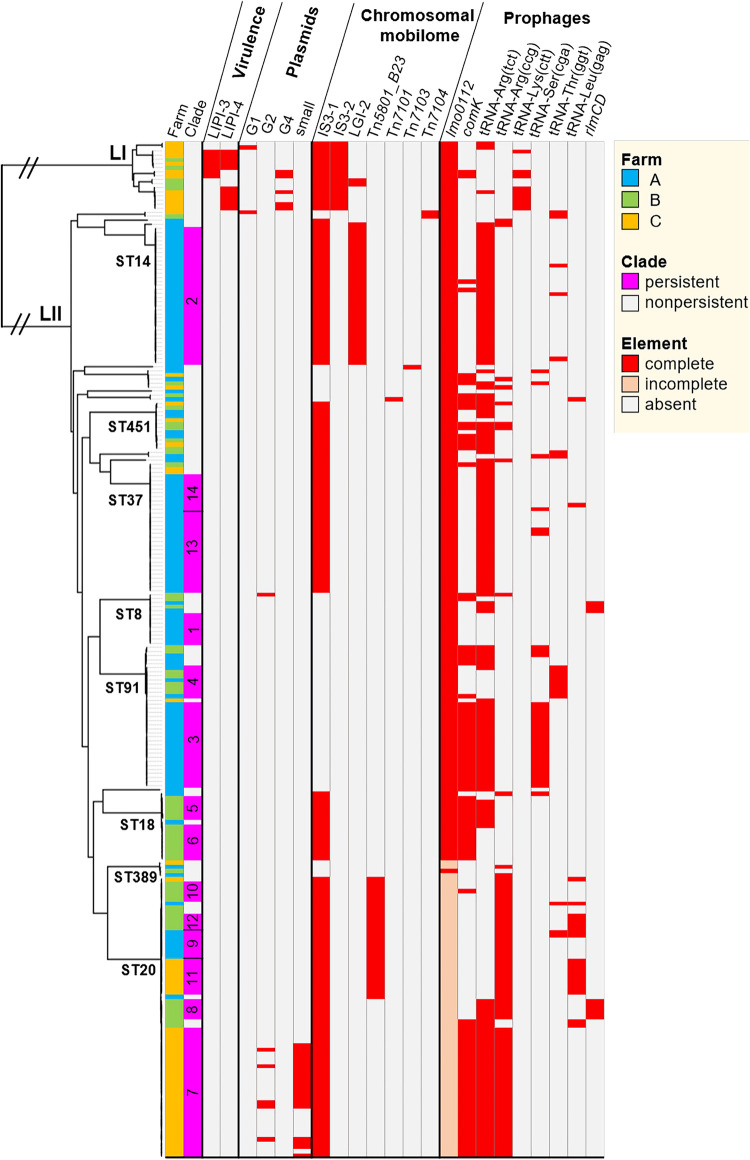
Phylogeny and genomic elements of 250 L. monocytogenes dairy farm isolates. The Lyve-SET 1.1.4f single-nucleotide polymorphism (SNP)-calling pipeline was used to generate an alignment file of the 250 genomes using L. monocytogenes EGD-e (GenBank accession number NC_003210.1) as a reference. Recombinant sites were removed from the alignment using Gubbins 3.0. Maximum-likelihood phylogeny was inferred from concatenated SNP alignment files using PhyML 3.3. The tree was visualized using FigTree 1.4.4. Pathogenicity islands, plasmids, chromosomally located mobile elements, and prophages were identified from the assembled and annotated draft genomes. The heatmap is restricted to genomic elements that were detected in this study. Persistent clade numbers corresponding to data in Table 1 are shown. Plasmids are categorized by phylogenetic group and prophages by insertion site. L, lineage; ST, multilocus sequence type; LIPI, *Listeria* pathogenicity island; IS*3*, *Listeria* IS*3*-like element; LGI-2, *Listeria* genomic island 2.

Whole-genome sequencing and subsequent *in silico* subtyping of 250 Listeria monocytogenes isolates, collected from three Finnish dairy farms during 2013 to 2016 (5), yielded 25 unique multilocus sequence types (STs) (Fig. 1a; see also Data Set S1 in the supplemental material). The most frequently detected subtype was ST20, which represented 28% of all sequenced isolates. In this study, persistent clades of L. monocytogenes were defined as monophyletic clades of isolates with pairwise distances (PWDs) of fewer than 20 SNPs (25) that were isolated from the same farm from ≥3 samples during ≥6 months. Clades that did not meet these criteria were classified as nonpersistent. In total, we identified 14 persistent clades (Fig. 2 and Table 1). Persistent clades represented 71% of all sequenced isolates, and all persistent clades belonged to serogroup 1/2a. Clade C4 contained isolates from two different farms, suggesting that strains of L. monocytogenes can spread between farms more quickly than the rate of genomic diversification.

**TABLE 1:** Pairwise distances within persistent clusters of L. monocytogenes from dairy farms A to C

Cluster	CC^*a*^	ST^*b*^	CT^*c*^	*N* ^*d*^	Farm(s)	Pairwise distance (no. of SNPs)		
						Mean	Minimum	Maximum
C1	8	8	9176	8	A	1.5	0	4
C2	14	14	9177	34	A	3.6	0	12
C3	14	91	9178	18	A	2.6	0	7
C4	14	91	9179	8	A, B	2	0	6
C5	18	18	9180	8	B	3.3	0	8
C6	18	18	9181	6	B	1.7	0	10
C7	20	20	9182	32	C	2.4	0	7
C8	20	20	9189	5	B	3.5	0	9
C9	20	20	9183	6	A	3.9	0	10
C10	20	20	9184	5	B	2.4	0	6
C11	20	20	9185	9	C	5.8	0	11
C12	20	20	9186	4	B	1.5	0	6
C13	37	37	9187	20	A	2.6	0	7
C14	37	37	9188, 9205	7	A	2.2	0	6
All clusters						2.8	0	8

a CC, clonal complex.

b ST, multilocus sequence typing (MLST) profile.

c CT, core genome multilocus sequence typing (cgMLST) profile.

d Number of isolates in the persistent cluster.

10.1128/mSphere.00383-21.1 DATA SET S1 Accession numbers, subtyping results, metadata, and mobile genetic elements of the 250 L. monocytogenes isolates sequenced and analyzed in this study. Download Data Set S1, XLSX file, 0.1 MB. Copyright © 2021 Castro et al. 2021 Castro et al. https://creativecommons.org/licenses/by/4.0/ This content is distributed under the terms of the Creative Commons Attribution 4.0 International license.

Pathogenicity islands associated with hypervirulence (*Listeria* pathogenicity island 3 [LIPI-3] and LIPI-4) were detected in 5% of the 250 isolates, none of which belonged to persistent clades. None of the 250 isolates harbored a premature stop codon within the *inlA* gene, which is associated with hypovirulence and is a common finding in L. monocytogenes from food processing environments (26). Indeed, the two STs most stringently associated with the food processing environment, namely, ST9 and ST121 (26), were not detected in this study.

### Mobile genetic elements were on average more numerous among isolates in persistent than in nonpersistent clades of L. monocytogenes.

**FIG 3:**
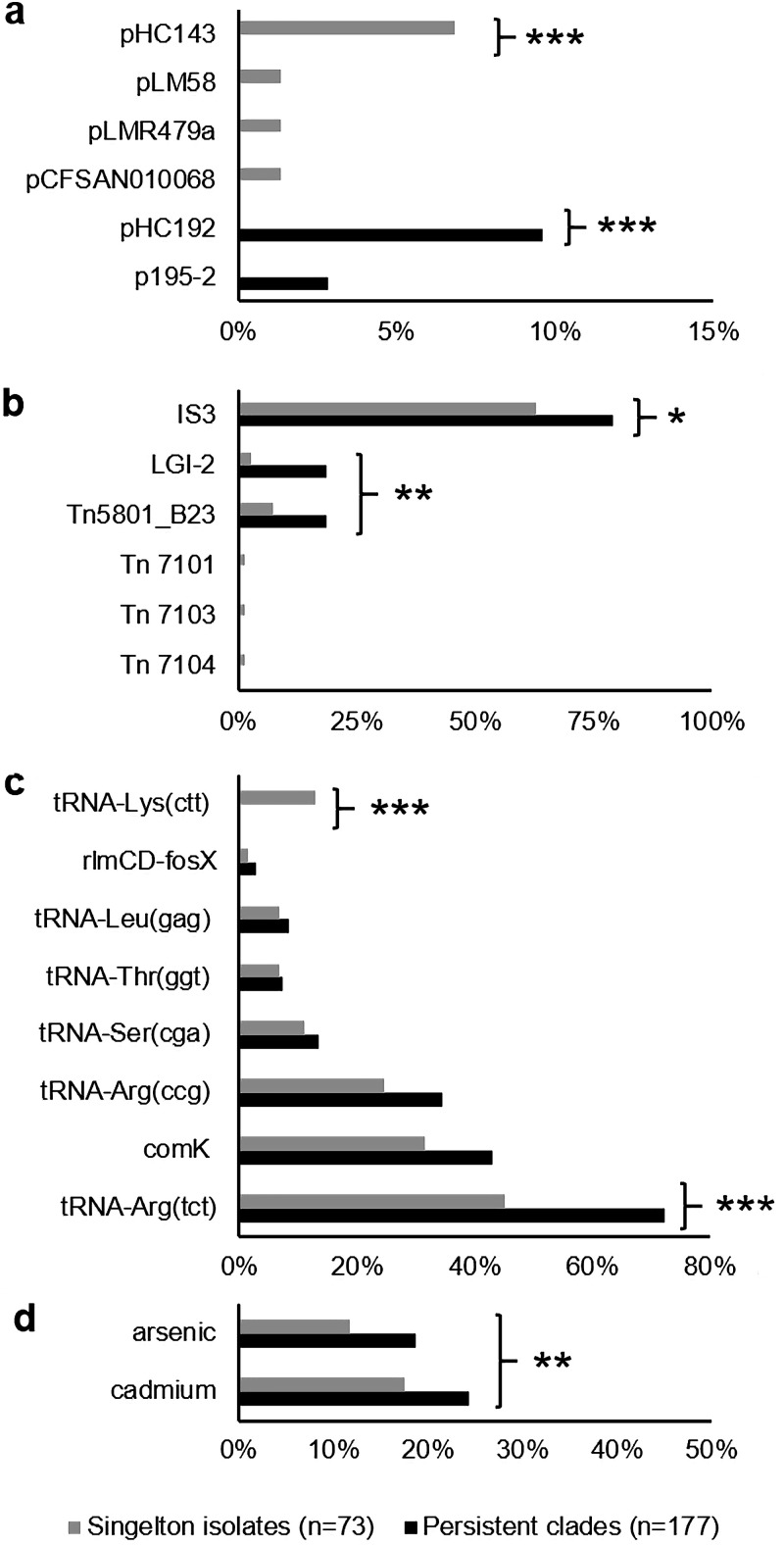
Occurrence of mobile genetic elements and heavy metal resistance genes among persistent and nonpersistent clades. Occurrence of plasmids (a), chromosomally located mobile elements (b), prophages (c), and cadmium and arsenic resistance genes (d) among isolates in persistent and nonpersistent clades. Significant differences between persistent clade isolates and singleton isolates are denoted by asterisks as follows: *, *P < *0.05; **, *P < *0.005; ***, *P < *0.001. IS*3*, *Listeria* IS*3*-like transposon; LGI-2: *Listeria* genomic island 2. Prophages are categorized by insertion site.

Overall, prophages and other mobile genetic elements were significantly more numerous among isolates in persistent than among those in nonpersistent clades (*P < *0.01; independent samples median test) (Fig. 1b and c). Resistance cassettes against cadmium and arsenic were detected in 20 and 15% of isolates, respectively. Mobile elements harboring resistance genes against arsenic and cadmium were significantly more prevalent among isolates in persistent clades than among those in nonpersistent clades (Fig. 3d). Surprisingly, 12% of all L. monocytogenes isolates harbored a putative bacitracin resistance cassette (27), located on the transposon Tn*5801*_B23. Other antimicrobial or biocide resistance genes were not detected in this study.

### Dairy farm isolates of L. monocytogenes harbored plasmids that are common in the food industry and three novel plasmids.

**FIG 4:**
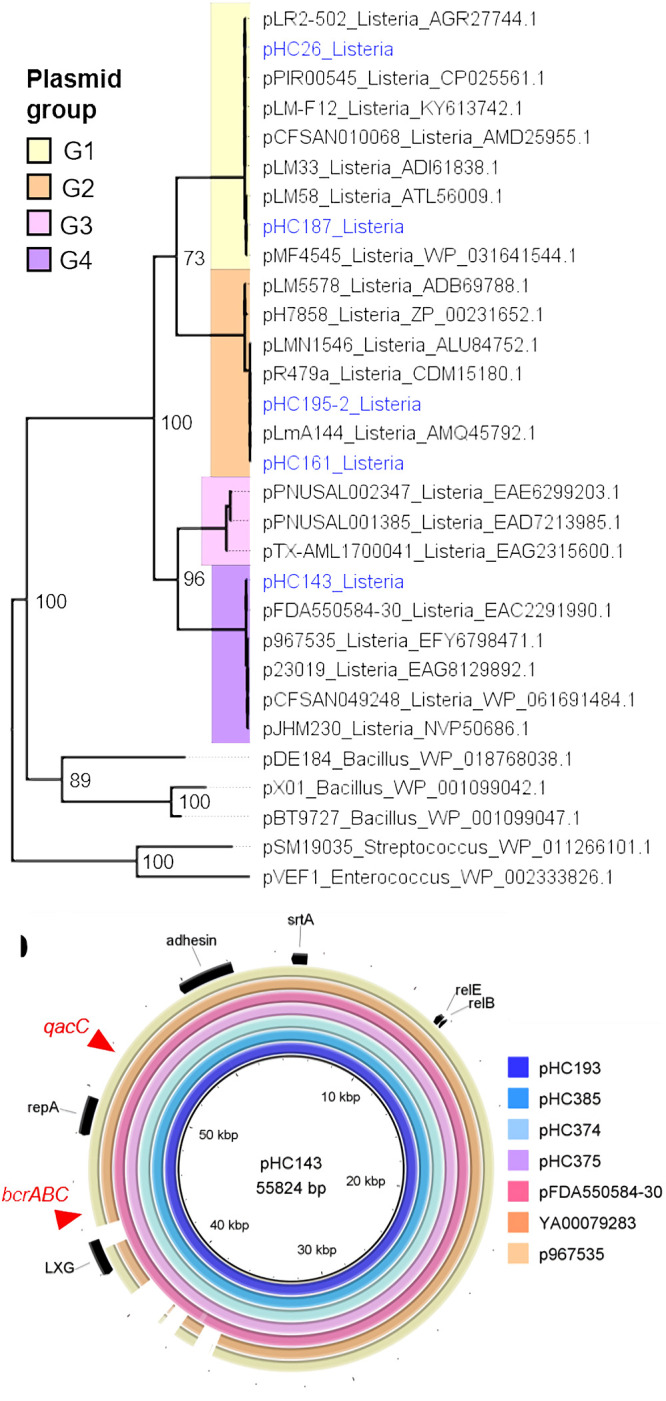
Characterization of plasmids based on RepA. (a) Maximum-likelihood phylogenetic analysis of the >50-kb plasmids detected in the present study, based on the *repA* amino acid sequences. The analysis employed the Jones-Taylor-Thornton substitution model with 100 bootstraps and was performed using MEGA7 software. Bootstrap support values above 70 are shown. Plasmids represented three phylogenetic clades (plasmid groups G1, G2, and G4). Plasmid groups correspond to the groups established by Kuenne et al. (28) and Schmitz-Esser et al. (29). Tip labels correspond to plasmid names and host genera; plasmids from this study are labeled in blue. (b) G4 plasmids of the L. monocytogenes strains HC193 (this study), HC374 (this study), and FDA550584-30 (BioSample accession number SAMN02923676) aligned with >95% identity across the entire length of pHC143 from this study; plasmids of the L. monocytogenes strains 967535 (BioSample accession number SAMN15680309) and YA00079283 (accession number SAMN08970420) aligned with >95% identity to most of pHC143. Red arrows indicate the insertion sites of the biocide resistance loci *qacC*, present in p967535 and pYA00079283, and *bcrABC*, present in pFDA550584-30. The alignment was generated using BRIG 0.95. For pHC143, plasmid length in base pairs (bp) is given.

Plasmids were detected among 10% of L. monocytogenes isolates in persistent clades and 11% of isolates in nonpersistent clades. We detected three previously identified plasmids (pCFSAN010068, pLM58, and pLMR479a) and three novel plasmids, which were labeled pHC143, pHC192, and pHC195-2 (Fig. 3a and Data Set S1). These plasmids were 55.5 to 86.7 kb in size, except for pHC192, which was only 4.6 kb. A maximum-likelihood phylogenetic analysis based on RepA grouped the five large plasmids into the plasmid groups G1, G2, and G4 (28, 29), which appear to be specific to the genus *Listeria* (Fig. 4a). Plasmid groups G1 and G2 include well-characterized L. monocytogenes reference plasmids that are common in food processing environments (18, 19, 28). G4 represents a novel group of *Listeria* plasmids (29).

The G4 plasmid pHC143 was detected in five isolates of this study, belonging to ST6 and ST149 (see Data Set S1). These STs are hypervirulent, based on the presence of pathogenicity islands LIPI-3 (ST6) and LIPI-4 (ST149) (Fig. 2). Visualization of assembly graphs indicated that pHC143 was successfully assembled into a single 55.8-kb contig in all five isolates. pHC143 contained no biocide or heavy metal resistance genes. However, we identified three variants of pHC143 among short-read sequence assemblies deposited in GenBank, all of which contain resistance genes against biocides (Fig. 4b; see also Fig. S1 in the supplemental material). The first variant contains a benzalkonium chloride resistance cassette (*bcrABC*) and a mercuric resistance (*mer*) operon. The second variant contains a multidrug exporter putatively conferring resistance against quaternary ammonium compounds (*qacC* [*qacH*]; GenBank accession number WP_000121134.1). The third variant contains the *qacC*/*qacH* gene and a Tn*554* family transposon carrying an arsenic resistance operon (*arsABCD*). This Tn*554* family transposon was identified previously in the chromosomes of L. monocytogenes (30). All G4 plasmids contained a predicted fimbrial adhesin (GenBank accession number WP_061691480.1), suggestive of a role associated with attachment and host colonization (31).

10.1128/mSphere.00383-21.2 FIG S1 Comparison of group 4 (G4) plasmids. Alignment of pFDA550584-30 (A), pHC143 (B), p967535 (C), pYA00079283 (D), and the Tn*554*-like transposon of the strain SLCC2372 (E). pFDA550584-30 contains mercury (*mer* operon) and benzalkonium chloride (*bcrABC*) resistance cassettes; p967535 and pYA00079283 contain the biocide resistance gene *qacC*. Additionally, pYA00079283 contains a transposon carrying the arsenic resistance cassette (*arsABCD*). Coloring reflects functional annotation; genes conserved in all G4 plasmids are colored in gray. Download FIG S1, PDF file, 0.2 MB. Copyright © 2021 Castro et al. 2021 Castro et al. https://creativecommons.org/licenses/by/4.0/ This content is distributed under the terms of the Creative Commons Attribution 4.0 International license.

**FIG 5:**
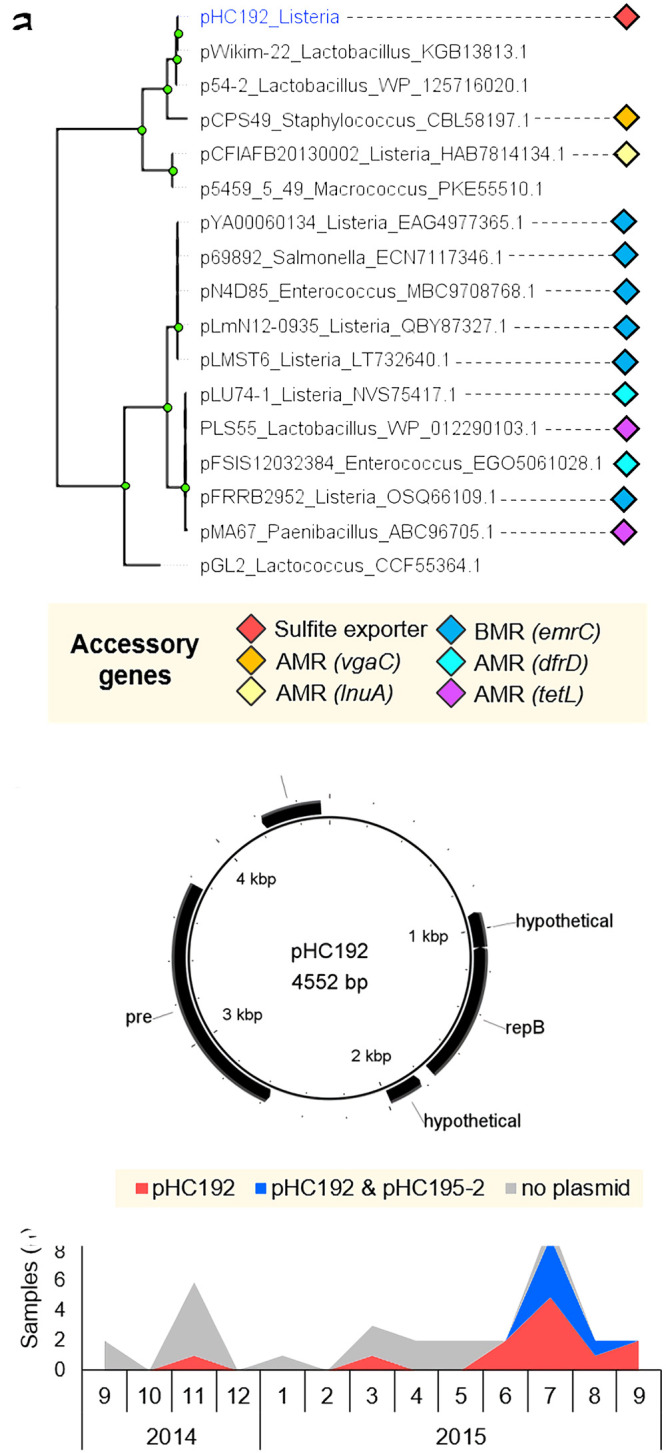
Phylogeny, gene content, and epidemiology of the novel plasmid pHC192. (a) Maximum-likelihood phylogenetic analysis of pHC192 and related plasmids, based on the *repB* amino acid sequences. Plasmids other than pHC192 were identified and obtained from GenBank using BLASTp. The analysis employed the Jones-Taylor-Thornton substitution model with 100 bootstraps and was performed using the MEGA7 software. Node labels indicate bootstrap support values above 70. Tip labels correspond to plasmid names and host genera; plasmids from this study are labeled in blue. Tip shapes depict harborage of resistance genes against antimicrobials (antimicrobial resistance [AMR]) and biocides (biocide resistance [BCR]). (b) The 4.5-kb plasmid pHC192, carrying a putative SafE/TauE family sulfite exporter (GenBank accession number WP_016896343.1). The figure was constructed using BRIG 0.95. Plasmid length in base pairs (bp) is given. (c) Numbers of samples containing no plasmid, pHC192, or both pHC192 and pHC195-2 among persistent clade C7 isolates during each month of sampling. Plasmid prevalence in C7 isolates increased over the 1-year sampling period.

Assembly graphs of the small plasmid pHC192 suggested that the plasmid was closed successfully into a single 4.6-kb contig. pHC192 did not contain replication proteins related to the RepA of *Listeria* plasmid groups G1 to G4, so the phylogeny of this plasmid was analyzed using RepB (Fig. 5a). Phylogenetically, pHC192 clustered closely with plasmids from *Lactobacillus*. Indeed, RepB of pHC192 (GenBank accession number WP_035147907.1) was also detected in Lactobacillus and Brochothrix (100% amino acid sequence identity), suggestive of a broad host range for this plasmid. The closest relative of pHC192 in *Listeria* was the plasmid of L. monocytogenes strain CFIAFB20130002, which possesses the lincosamide resistance gene *lnuA* (GenBank accession number WP_001829870.1). Notably, RepB of pHC192 bore no similarity to the replication proteins of the small *Listeria* plasmids pIP823 (GenBank accession number WP_172694646.1) and pDB2011 (accession number WP_020277964.1) and shared only 45% amino acid identity with the RepB of pLMST6 (accession number WP_061092472.1). Like pHC192, pLMST6 appears to also have a broad host range, as 100% identical homologues of pLMST6 RepB (accession number WP_061092472.1) were detected in *Listeria*, Salmonella, and Enterococcus. These findings suggest that several phylogenetically unrelated small plasmids have been acquired by *Listeria* through distinct transfer events across host species.

The plasmid pHC192 contains a putative *tauE* (*safE*) family sulfite exporter gene (GenBank accession number WP_016896343.1) (Fig. 5b) that is not typically present in *Listeria* plasmids (19, 29). The sequencing depth of coverage for pHC192 was approximately five times that of the chromosome, suggesting that pHC192 is a high-copy-number plasmid. This plasmid became increasingly prevalent among persistent clade C7 isolates during the sampling period and was detected in all isolates at the end of the study (Fig. 5c). An additional plasmid, pHC195-2, was detected in several isolates of clade C7 in the latter part of the sampling period. The pHC195-2 plasmid belonged to the phylogenetic group G2 (Fig. 4a) and closely resembled the reference plasmid pLMR479a (see Fig. S2 in the supplemental material). The acquisition of these plasmids during the course of persistence suggests that they play a role in the adaption of the pathogen to the farm ecosystem.

10.1128/mSphere.00383-21.3 FIG S2 Plasmids of groups G1 and G2 detected in this study. Plasmids identical to pLMR479a (a), pCFSAN010068 (b), and pLM58 (c) were detected in this study. Plasmid pHC195-2 was identical to pLMR479a, except for the absence of a putative DEAD/DEAH box helicase (GenBank accession number WP_077913968). Genes putatively associated with heavy metal detoxification or stress tolerance are shown. Download FIG S2, PDF file, 0.1 MB. Copyright © 2021 Castro et al. 2021 Castro et al. https://creativecommons.org/licenses/by/4.0/ This content is distributed under the terms of the Creative Commons Attribution 4.0 International license.

### Dairy farm isolates of L. monocytogenes share common integrative mobile elements with enterococci.

Among the 250 dairy farm isolates, we identified the following six chromosomally located mobile elements: the L. monocytogenes IS*3*-like element (30); *Listeria* genomic island 2 (LGI-2) (32); Tn*5801*_B23 (33); and three novel mobile elements, which were submitted to the Transposon Registry (34) and assigned the labels Tn*7101*, Tn*7103*, and Tn*7104*. The elements ICELm1 (30), LGI-1 (35), LGI-3 (36), Tn*554* (30), Tn*6188* (20), Tn*6198* (37), and chromosomally located Tn*5422* (38) were not detected.

The IS*3*-like transposon was significantly more prevalent among isolates in persistent than in nonpersistent clades (Fig. 3b). The IS*3*-like transposon consists of two insertion sequences in lineage I (IS*3-1* and IS*3-2*) and a single insertion sequence in lineage II (IS*3-1*) (Fig. 2). These elements harbor multiple surface-associated lipoproteins, which may facilitate attachment and invasion (30). The suggested role of the IS*3*-like transposon in L. monocytogenes virulence remains to be determined.

The integrative and conjugative elements (ICEs) LGI-2 and Tn*5801*_B23 were significantly more prevalent among isolates in persistent than in nonpersistent clades (*P < *0.01; Fisher’s exact test) (Fig. 3b). LGI-2 carries cadmium and arsenic resistance cassettes and two multidrug transporters (see Fig. S3 in the supplemental material). Identical (100% nucleotide identity) LGI-2 elements were present among all ST14 and ST145 isolates in this study (Fig. 2). Moreover, a BLASTn search identified identical LGI-2 elements in 11 L. monocytogenes and two Enterococcus faecalis complete genomes, suggestive of recent transfer between these species.

10.1128/mSphere.00383-21.4 FIG S3 *Listeria* genomic island 2 (LGI-2), Tn*7101*, and Tn*7102* were identical in Listeria and Enterococcus. LGI-2 was identical in sequence type 14 (ST14) (A) and ST145 (B) of this study, in *Listeria* strain J1-220 (C), and in *Enterococcus* strain 110 (D). Tn*7102* was identical in *Listeria* strain ICDC_LM1233 (E) and *Enterococcus* strain H112E (F). Tn*7101* was identical in strain HC258 of this study (G) and *Enterococcus* VE80 (H). LGI-2 is an integrative and conjugative element containing an integrase and a type IV secretion system. The novel elements Tn*7101* and Tn*7102* contain ICEBs1_C-like integrases but lack the conjugation infrastructure. Red outlining indicates pseudogenes. Download FIG S3, PDF file, 0.2 MB. Copyright © 2021 Castro et al. 2021 Castro et al. https://creativecommons.org/licenses/by/4.0/ This content is distributed under the terms of the Creative Commons Attribution 4.0 International license.

Tn*5801*_B23 was detected in a subset of ST20 isolates, including the persistent clades C9 to C12 (Fig. 2 and Data Set S1). The Tn*5801*_B23 detected in this study shared 97% nucleotide identity with the Tn*5801*_B23 of Enterococcus faecalis strain JH2-2 (see Fig. S4 in the supplemental material). Tn*5801*_B23 contains putative resistance genes against the antimicrobial bacitracin (*bcrABD*) and a two-component system (*baeSR*) potentially involved in the regulation of the *bcrABD* operon (33). Unlike Tn*5801*_B23, other Tn*5801*-like elements mediate tetracycline resistance in *Enterococcus*, *Listeria*, and several other Firmicutes species (33). In L. monocytogenes ST20, Tn*5801*_B23 was inserted downstream of *guaA* (*lmo1096*), which is also the insertion site of the related element ICELm1 of L. monocytogenes strain EGD-e, harboring cadmium resistance genes (30).

10.1128/mSphere.00383-21.5 FIG S4 Comparison of Tn*5801*-like integrative and conjugative elements. Tn*5801*_B23 is identical in *Enterococcus* JH2-2 (A) and the ST20 strains of this study (B). Tn*5801*_B23 contains a bacitracin resistance cassette, *bcrABD*. Tn*5801*_B23 is related to Tn*5801*_B15 from *Enterococcus* Ef1 (C), to the Tn*5801*_B15-like transposon of *Listeria* L2624 (D), and to ICELm1 from *Listeria* EGD-e (E). Download FIG S4, PDF file, 0.2 MB. Copyright © 2021 Castro et al. 2021 Castro et al. https://creativecommons.org/licenses/by/4.0/ This content is distributed under the terms of the Creative Commons Attribution 4.0 International license.

The putative integrative and mobilizable element (IME) Tn*7101* was detected in the ST155 singleton isolate HC258, where it was inserted between homologues of *lmo2596* and *lmo2597* (see Fig. S3). Tn*7101* contains resistance genes against cadmium (*cadA* and *cadC*) and an arsenate reductase (*arsC*). Through a BLASTp search, we identified a variant of Tn*7101* containing a seven gene arsenic resistance cassette. This variant, labeled Tn*7102*, was detected in several L. monocytogenes and *Enterococcus* genomes deposited in GenBank (see Fig. S2). The Tn*7101* and Tn*7102* of *Listeria* and Enterococcus were identical (100% nucleotide identity), suggestive of recent promiscuity between the two genera. Arsenic resistance genes in Tn*7102* were distantly related (≥67% nucleotide identity) to the arsenic resistance cassette of LGI-2 (see Fig. S3).

The putative IME Tn*7103* was detected in the ST119 singleton isolate HC183, where it was inserted between *lmo0810* and *lmo0811*. This transposon contained putative virulence genes encoding an InlJ-like internalin and a bacterial immunoglobulin (Big)-like protein (see Fig. S5 in the supplemental material). A BLAST search confirmed the presence of Tn*7103* in other L. monocytogenes strains, including N12-2532 (BioSample accession number SAMN09947958), but we did not identify this element in other species.

10.1128/mSphere.00383-21.6 FIG S5 Comparison of Tn*7103.* Tn*7103* of the L. monocytogenes strain N12-2532 (A) was identical to HC183 from the present study (B). Tn*7103* is a novel integrative and conjugative element encoding internalin J-like and bacterial immunoglobulin 3-like (Big-3) proteins, putatively associated with attachment and invasion. Download FIG S5, PDF file, 0.1 MB. Copyright © 2021 Castro et al. 2021 Castro et al. https://creativecommons.org/licenses/by/4.0/ This content is distributed under the terms of the Creative Commons Attribution 4.0 International license.

The putative ICE Tn*7104* was detected in the ST391 singleton isolate HC187 and was inserted between *lmo1786* and *lmo1787.* This transposon contained a putative type I restriction modification system (see Fig. S6 in the supplemental material). Tn*7104* was identified in several other L. monocytogenes strains deposited in GenBank, including the L. monocytogenes ST391 strain SHL013 (BioSample accession number SAMN03265960), but we did not identify this element in other species.

10.1128/mSphere.00383-21.7 FIG S6 Comparison of Tn*7104.* Tn*7104* of the L. monocytogenes ST391 strain SHL013 (A) was identical to that of the ST391 strains of the present study (B). Tn*7104* is a novel mobile element encoding a putative type I restriction-modification system. Tn*7104* contains an integrase but appears to lack a type IV secretion system, suggesting that it is an integrative and mobilizable element (IME). Red bordering of a gene indicates that it is a pseudogene. Download FIG S6, PDF file, 0.1 MB. Copyright © 2021 Castro et al. 2021 Castro et al. https://creativecommons.org/licenses/by/4.0/ This content is distributed under the terms of the Creative Commons Attribution 4.0 International license.

### A novel prophage harboring cadmium resistance genes was identified in a persistent clade of L. monocytogenes.

All 250 dairy farm isolates from this study contained the L. monocytogenes monocin (39) and 0 to 3 additional prophages, which were detected at eight insertion sites (Fig. 3c). Prophages inserted into tRNA-Arg(tct) were significantly more prevalent among isolates in persistent clades, and prophages inserted into tRNA-Lys(ctt) were significantly more prevalent among nonpersistent clades (*P < *0.05; Fisher’s exact test).

OPTSIL taxonomic clustering assigned prophages from this study into six genera. Prophages inserted into *comK* and tRNA genes were assigned to genera of Siphoviridae that are known to only infect *Listeria*. Surprisingly, in the isolate HC189, a 67-kb Myovirus was inserted into *comK*, a site usually occupied by *Siphoviridae* (40).

**FIG 6:**
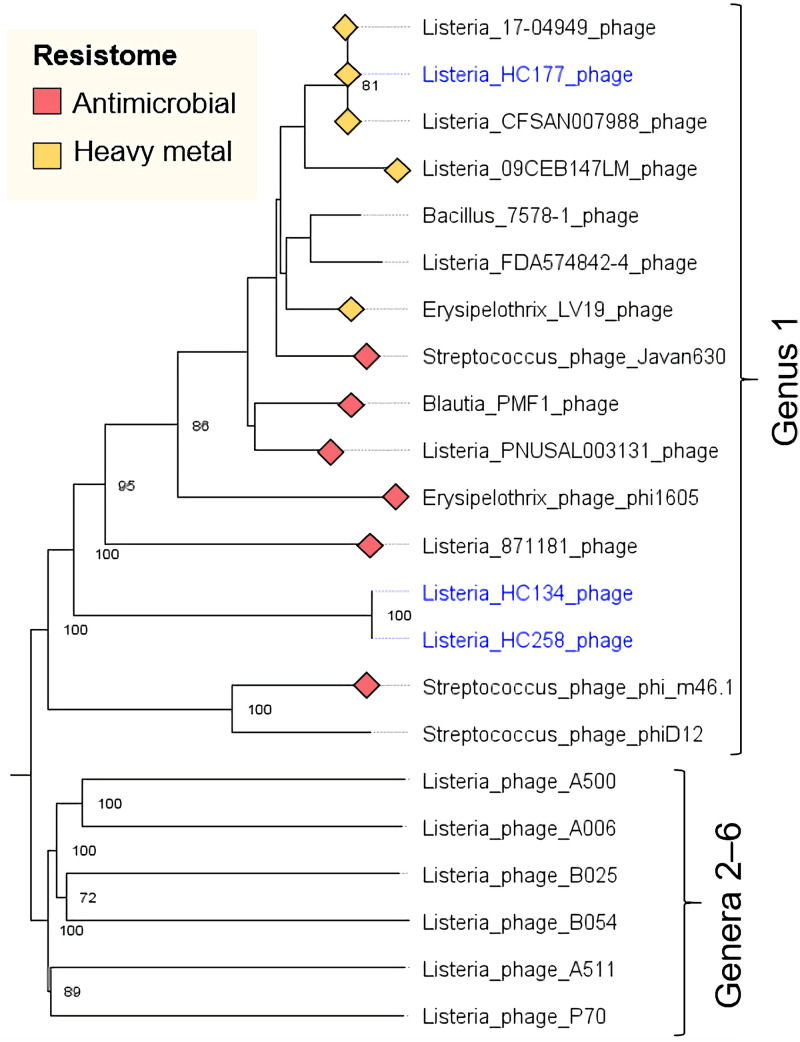
Prophages inserted between *rlmCD* and *fosX* belonged to the genus *Siphovirus* and have a broad host species range and a tendency to harbor antimicrobial or heavy metal resistance genes. Minimum evolutionary tree and taxonomic clustering of six *Listeria*-specific phages (genera 2 to 6), prophages from the strains HC134 and HC258 from this study that were inserted between *rlmCD* and *fosX* (genus 1, blue), and related prophages from *Listeria* and other *Firmicutes* species obtained from GenBank (genus 1, black). Phylogenetic analyses and clustering were generated with the VICTOR online tool (https://victor.dsmz.de), using model D_6_ and 100 bootstrap replicates. The tree was visualized using FigTree 1.4.4. Bootstrap support values above 70 are shown.

Prophages inserted between the *rlmCD* (*lmo1703*) and *fosX* (*lmo1702*) genes were not related to any of the *Listeria*-specific phage genera, but instead represented a separate genus that infects several *Firmicutes* species (Fig. 6). Many of the phages in this genus harbor antimicrobial and heavy metal resistance cassettes (see Fig. S7 in the supplemental material). In this study, phages inserted between *rlmCD* and *fosX* were detected among all isolates of persistent clade C8 and among three singleton isolates (Fig. 2). Among isolates of persistent clade C8, prophages inserted between *rlmCD* and *fosX* all harbored a cadmium resistance cassette (see Fig. S7). In contrast, in the singleton isolates, prophages inserted between *rlmCD* and *fosX* harbored no cadmium or antimicrobial resistance genes. Within *Listeria* genomes deposited in GenBank, we identified prophages inserted between *rlmCD* and *fosX* that carried resistance genes against cadmium (*cadA*), macrolides (*mefA*, *msrD*), tetracycline (*tetM*), and streptogramin (*vatA*).

10.1128/mSphere.00383-21.8 FIG S7 Antimicrobial and heavy metal resistance genes carried by genus 1 prophages. Schematic representation of the antimicrobial and heavy metal resistance cassettes carried by genus 1 prophages. The amino acid (aa) length is written below each gene; the gene name or domain is written on top. Gene colors reflect functional prediction. Isolates form this study are labeled in blue. Download FIG S7, PDF file, 0.2 MB. Copyright © 2021 Castro et al. 2021 Castro et al. https://creativecommons.org/licenses/by/4.0/ This content is distributed under the terms of the Creative Commons Attribution 4.0 International license.

### Systems that protect against invading DNA were negatively associated with the persistence of L. monocytogenes on dairy farms.

A genome-wide association study was conducted to assess which genes were associated with persistent versus nonpersistent clades. Because no persistent clades belonged to lineage I, the analysis was restricted to lineage II. Notably, a gene involved in biofilm formation (*bapL*), which has been implicated in the adaptation of L. monocytogenes to the food processing environment (22), was significantly associated with persistence on dairy farms (see Table S2 in the supplemental material). In contrast, genes associated with the CRISPR-*cas* type IIA system and the type II restriction-modification system LmoJ3 (24) were negatively associated with nonpersistence (see Table S2). CRISPR-*cas* systems and restriction modification systems may act in synchrony to protect the host against invading prophages and other mobile elements (41). Additionally, a putative recombination and DNA strand exchange inhibitor protein (GenBank accession number WP_031664941.1) was associated with nonpersistence. These findings agree with the lower prevalence of mobile genetic elements and prophages among isolates in nonpersistent than those in persistent clades and suggest that systems involved with inhibiting invading DNA are detrimental for the persistence of L. monocytogenes in the dairy farm environment.

10.1128/mSphere.00383-21.10 TABLE S2 Genes positively or negatively associated (Bonferroni-corrected *P* < 0.05) with persistent clades among the 233 L. monocytogenes lineage II isolates included in this study. Download Table S2, PDF file, 0.1 MB. Copyright © 2021 Castro et al. 2021 Castro et al. https://creativecommons.org/licenses/by/4.0/ This content is distributed under the terms of the Creative Commons Attribution 4.0 International license.

Curiously, the ESX-1-like type VII secretion system (T7SS) contained both genes associated with persistence and genes associated with nonpersistence. The T7SS of L. monocytogenes has a potential role in bacterial antagonism (42) and is located in L. monocytogenes hypervariable hot spot 1 (30). Overall, many of the genes associated with persistence or nonpersistence belonged to L. monocytogenes hypervariable hot spots or prophages, suggesting that the role these components play in *Listeria* niche adaptation deems further study.

## DISCUSSION

Whole-genome sequencing and subsequent analyses of 250 L. monocytogenes isolates from dairy farms illustrated that dairy farm isolates are hosts to a diversity of mobile genetic elements that carry, or have the potential to carry, resistance genes against antimicrobials, biocides, and heavy metals. Many of the mobile elements we identified carried genes encoding phenotypes that promote the survival of L. monocytogenes on farms, such as antimicrobial resistance genes or virulence factors. Moreover, genes responsible for the conjugation of mobile elements may have a dual role in promoting biofilm formation and invasion of the mammalian host (31, 43), further enhancing the survival of L. monocytogenes on farms. We found that prophages and other mobile genetic elements were significantly more numerous among isolates belonging to persistent than nonpersistent clades. Moreover, systems that provide immunity against invading mobile genetic elements (23, 24, 41), namely, the CRISPR-*cas* IIA system, the type II restriction modification system LmoJ3, and a putative recombination and DNA strand exchange inhibitor protein, were associated with nonpersistence. Together, these findings suggest that mobile elements may support the persistence of L. monocytogenes inhabiting farms.

Most of the mobile genetic elements we uncovered appeared in a very limited number of L. monocytogenes STs. The narrow distribution and wide diversity of the mobile genetic elements we identified likely explain why very few mobilome-associated genes were significantly associated with persistence in this study. As an exception, the IS*3*-like element of L. monocytogenes (30) and prophages at certain insertion sites were widely distributed across STs and harbored genes that were significantly associated with either persistence or nonpersistence.

We identified a surprising diversity of mobile genetic elements encoding heavy metal resistance genes among the dairy farm isolates. Moreover, acquired heavy metal resistance genes were more common among isolates in persistent than nonpersistent clades. Heavy metal resistance is also more common among persistent than nonpersistent L. monocytogenes subtypes from foods and food processing environments (14, 16). Whether heavy metal resistance genes contribute directly to persistence or merely cooccur with other determinants that promote environmental survival remains unclear (44). Nevertheless, heavy metal resistance genes may represent useful markers to aid the detection of L. monocytogenes strains with enhanced resilience against environmental stressors.

In the present study, we found a novel plasmid (pHC143; plasmid group G4) that infected hypervirulent subtypes of L. monocytogenes. Although pHC143 was devoid of biocide and heavy metal resistance genes, such genes are common on other G4 plasmids infecting hypervirulent ST1 and ST6 strains (29). Indeed, we noted a G4 plasmid harboring the biocide resistance gene *qacC* and the arsenic resistance cassette *arsABCD* in the ST6 outbreak isolate YA00079283, associated with the largest listeriosis outbreak known to date (45). It is plausible that harborage of biocide and heavy metal resistance genes in G4 plasmids facilitates the adaptation of hypervirulent strains to food processing environments.

We identified four transposons in *Listeria*, namely, LGI-2, Tn*5801*_B23, Tn*7101*, and Tn*7102*, that closely resembled transposons in *Enterococcus*, suggestive of recent transfer between the two genera. The cooccurrence of genomic elements in *Enterococcus* and *Listeria* was unsurprising, as both genera are highly prevalent in animal feces and farms (5, 8, 46). Transfer of conjugative elements has been demonstrated both from *Enterococcus* to *Listeria* and vice versa (47, 48) indicating that both genera are potential donors. The extent to which enterococci and other *Firmicutes* contribute to the horizontal spread of mobile elements and their associated antimicrobial, biocide and heavy metal resistance determinants in *Listeria* has implications for food safety and should be explored through further study.

Bacitracin resistance genes, mediated by Tn*5801*_B23, were common among L. monocytogenes isolates from all three farms investigated. Moreover, Tn*5801*_B23 was significantly more prevalent among isolates in persistent clades than among those in nonpersistent clades. The widespread use of bacitracin as a growth promoter in animal feeds has facilitated the expansion of bacitracin resistance in *Enterococcus* (49, 50), and probably also in L. monocytogenes, as animal feeds are frequently contaminated by *Listeria* (5, 8). Nevertheless, the frequent detection of Tn*5801*_B23 in this study remains curious, as feed supplementation with bacitracin subsided in Finland in the 1990s (49).

We found that prophages were more prevalent among isolates in persistent clades than among those in nonpersistent clades. Whether prophages contribute to the persistence of L. monocytogenes is an intriguing possibility. There is increasing evidence that prophages can mediate beneficial phenotypes for their host. Phages mediate resistance or virulence properties in numerous bacterial species (51), and in *Listeria*, siphoviruses inserted into *comK* were found to regulate the gene in a symbiotic manner (40). Here, we discovered phage-mediated carriage of cadmium resistance and various antimicrobials in *Listeria*, suggesting that prophages contribute to the spread of phenotypes supporting persistence. Moreover, we noted that these phages belonged to a genus of Siphovirus with an apparently broad host species range that were introduced to *Listeria* through several distinct transfer evets. Host species jumps have the potential accelerate the transfer of novel resistance determinants between *Listeria* and other *Firmicutes*.

It is worth noting that not all persistent clades harbored mobile elements, suggesting that other factors also contribute to the survival of L. monocytogenes on dairy farms. We found that genes putatively involved in biofilm formation (*bapL*) and interbacterial competition (T7SS), which are not located in mobile elements, were significantly associated with persistence. In addition, the predominance of persistent L. monocytogenes strains in the dairy farm environment is associated with inadequacies in production hygiene (5). Therefore, the persistence of L. monocytogenes in the dairy farm environment is likely the result of a multifactorial combination of bacterial and environmental factors.

In conclusion, our study indicates that L. monocytogenes strains inhabiting the dairy farm environment are receptive to a diversity of prophages and mobile genetic elements. We suggest that mobile elements enable L. monocytogenes to adapt to the stresses encountered in the farm ecosystem and in general improve the fitness of the pathogen on farms, thereby supporting persistence. Given the abundance of L. monocytogenes on farms (2, 3, 5) and the apparent exchange of mobile genetic elements between *Listeria* and other *Firmicutes* species, L. monocytogenes occurring in agroecosystems should be viewed as a potential reservoir of mobile genetic elements. Importantly, many of these elements have the potential to carry and spread antimicrobial, biocide, and heavy metal resistance genes. The spread of mobile genetic elements and resistance determinants from primary production to *Listeria* in food processing environments has important food safety implications and should be explored further. The present study represents a step forward in this effort and in our understanding of listerial ecology in the agroecosystem.

## MATERIALS AND METHODS

### Whole-genome sequencing.

In total, 250 L. monocytogenes isolates obtained from three Finnish dairy cattle farms during 2013 to 2016 (5) were selected for whole-genome sequencing in the present study (see Data Set S1 in the supplemental material). The isolates were obtained from samples of bulk tank milk (31 isolates), used milk filters (46 isolates), feed (14 isolates), cow feces (21 isolates), and bedding materials (9 isolates) and from surface swab samples of floors (54 isolates), feed and water troughs (54 isolates), udders and udder cloths (10 isolates), milking systems and bulk tanks (8 isolates), stall mats (2 isolates), and strip cups (1 isolate). DNA was extracted from overnight cultures using the guanidium thiocyanate extraction method (52). DNA samples were standardized to a concentration of 10 ng/μl using the double-stranded DNA (dsDNA) broad-range (BR) assay kit (Thermo Fisher Scientific, Waltham, MA) using a Qubit fluorometer (Thermo Fisher Scientific). Genomic libraries were constructed from the DNA samples using the Nextera XT DNA sample preparation kit (Illumina, San Diego, CA), and paired-end sequencing (2 × 250 bp) was performed using the Illumina HiSeq 2500 platform.

### Genome assembly, pangenome construction, and subtyping.

Following the removal of adapter sequences and low-quality reads using Trimmomatic 0.36 (53), draft genomes were assembled using SPAdes 3.9 with *k*-mer values of 55, 77, 99, 113, and 127 (54). Assembly quality was assessed using QUAST 4.0 (55), and taxonomic assignment was performed using Kraken (56). The assemblies were annotated using Prokka 1.12 (57). The pangenome of the sequenced isolates was constructed using Roary 3.8.0 (58) with the protein identity cutoff value set at 90%. Multilocus sequence types (ST), corresponding to the schema developed by Ragon et al. (59), and core genome sequence types (CT), corresponding to the schema developed by Moura et al. (60), were determined *in silico* from the assembled genomes using the BIGSdb-*Lm* database. The BIGSdb-*Lm* database was also used to identify pathogenicity islands associated with hypervirulence (LIPI-3 and LIPI-4) and genes associated with antimicrobial and biocide resistance among the assembled genomes. Genome assemblies were deposited in GenBank under BioProject accession number PRJNA704814 (see Data Set S1).

### Maximum-likelihood phylogenomic analysis.

Phylogenomic reconstruction of the 250 L. monocytogenes isolates was performed using the Lyve-SET 1.1.4f pipeline (61), using the L. monocytogenes EGD-e genome (GenBank accession number NC_003210.1) as a reference. The Lyve-SET pipeline was run using Listeria monocytogenes presets (61), with the additional options “-mask-phages,” “-mask-cliffs,” and “-read_cleaner CGP.” In brief, the pipeline generated genome alignments by mapping quality-filtered reads to a reference genome. To improve the accuracy of phylogenomic inference, putative prophage genes were removed from the reference genome prior to mapping. Mapping was followed by the detection of high-quality SNPs, defined as having ≥10× depth of coverage and ≥75% consensus among reads. Recombinant sites within the genome alignments generated by Lyve-SET were identified and removed using Gubbins 3.0 (62). PhyML 3.3 (63) was used to infer maximum-likelihood phylogeny of each ST using a general time-reversible model (GTR) with 100 bootstrap replicates.

In addition, the phylogeny of each ST harboring putative persistent clades was inferred independently. Persistent clades of L. monocytogenes were defined as monophyletic clades of isolates with PWDs of <20 SNPs (25) that were isolated from the same farm from ≥3 samples during ≥6 months. For each ST, a draft assembly from the present study with the best quality statistics, i.e., the highest *N*_50_ value and lowest number of contigs (see Data Set S1), was used as a reference genome. The phylogenomic analyses were executed as described above using the Lyve-SET pipeline, Gubbins, and PhyML.

### Detection and analysis of plasmids.

Plasmids were identified by aligning the whole-genome assemblies against *Listeria* plasmids deposited in GenBank with the aid of BLASTn (http://www.ncbi.nlm.nih.gov/blast). Alignments were inspected manually. Additionally, whole-genome assembly graphs generated by SPAdes were visualized using Bandage 0.8.1 (64), and extrachromosomal elements were inspected manually. Maximum-likelihood phylogeny of the plasmids, based on the amino acid sequence alignments of the *repA* gene, was generated with MEGA7 (65), using the Jones-Taylor-Thornton substitution model with 100 bootstraps. Alignments of the amino acid sequences of the *repB* gene were used to compare plasmids in which *repA* was absent. Plasmid alignments were generated and visualized using BRIG 0.95 (66) and EasyFig 1.2 (67).

### Detection and analysis of chromosomal mobile genetic elements.

Draft assemblies from this study were screened for the presence of the mobile genetic elements ICELm1 (30), LGI-1 (35), LGI-2 (32), LGI-3 (36), Tn*5422* (37), Tn*6188* (20), and Tn*6198* (38), and the IS*3*-like and Tn*554*-like transposons of L. monocytogenes (30) by aligning the integrases, transposases, and recombinases associated with these elements against the pangenome (the “pan_genome_reference” file generated by Roary) with the aid of tBLASTn. Hits were inspected manually. Additionally, the pangenome was searched for annotations that included “recombinase,” “integrase,” “transposase,” “transposon,” “cadmium,” “arsenic,” “mercuric,” “*ardA*,” “*ftsK*,” “P60,” and “*iap*,” and hits were inspected manually. EasyFig 1.2 was used to align and visualize the identified transposons, and their occurrence among genomes deposited in GenBank was assessed using BLAST.

### Detection and analysis of prophages.

Prophages inserted into the L. monocytogenes genomes were identified using PHASTER (68), and the insertion sites were inspected manually. Phylogeny and taxonomic clustering of prophages classified by the PHASTER algorithm as “intact” were inferred using VICTOR (69). Nineteen additional *Listeria* phage genomes and one streptococcal phage genome obtained from GenBank were included in the analyses for reference (see Table S1). In brief, VICTOR applies the genome BLAST distance phylogeny (GBDP) method (70) to obtain pairwise distances, from which balanced minimum evolution trees are inferred. VICTOR utilizes OPTSIL (71) to obtain taxonomic clustering. Duplicate phage genomes are removed from the analysis. Trees generated by VICTOR were visualized using FigTree 1.4.4 (http://tree.bio.ed.ac.uk/software/figtree/). BLAST was used to identify phages inserted between *rlmCD* and *fosX* in the genomes of *Listeria* and other bacterial species deposited in GenBank, and hits were inspected manually. Phylogeny and taxonomic clustering of prophages inserted between *rlmCD* and *fosX* were inferred using VICTOR.

10.1128/mSphere.00383-21.9 TABLE S1 Phage genome sequences obtained from GenBank that were included the phylogenetic and taxonomic analysis of prophages. Download Table S1, PDF file, 0.04 MB. Copyright © 2021 Castro et al. 2021 Castro et al. https://creativecommons.org/licenses/by/4.0/ This content is distributed under the terms of the Creative Commons Attribution 4.0 International license.

### Identification of genes associated with predominance.

Scoary 1.6.16 (72) was used to identify genes that are significantly associated with occurrence in persistent versus nonpersistent clades. Scoary was executed using default options, using the “gene_presence_absence.csv” file generated by Roary as the input. Associations with a Bonferroni-corrected *P* value of 0.05 were considered significant. As all persistent clades belonged to lineage II, the analysis was limited to the 233 lineage II isolates of this study to reduce noise arising from population structure bias.

### Data availability.

The 250 L. monocytogenes isolates sequenced and analyzed in this study have been deposited in the NCBI BioSample database under accession numbers SAMN18056206 to SAMN18056455 and in GenBank under BioProject accession number PRJNA704814 and are described further in Data Set S1 in the supplemental material.

## References

[1] Gray MJ, Freitag NE, Boor KJ. 2006. How the bacterial pathogen *Listeria monocytogenes* mediates the switch from environmental Dr. Jekyll to pathogenic Mr. Hyde. Infect Immun 74:2505–2512. doi:10.1128/IAI.74.5.2505-2512.2006. 16622185 PMC1459693

[2] Nightingale KK, Schukken YH, Nightingale CR, Fortes ED, Ho AJ, Her Z, Grohn YT, McDonough PL, Wiedmann M. 2004. Ecology and transmission of *Listeria monocytogenes* infecting ruminants and in the farm environment. Appl Environ Microbiol 70:4458–4467. doi:10.1128/AEM.70.8.4458-4467.2004. 15294773 PMC492327

[3] Esteban JI, Oporto B, Aduriz G, Juste RA, Hurtado A. 2009. Faecal shedding and strain diversity of *Listeria monocytogenes* in healthy ruminants and swine in Northern Spain. BMC Vet Res 5:2–10. doi:10.1186/1746-6148-5-2. 19133125 PMC2651128

[4] Ho AJ, Lappi VR, Wiedmann M. 2007. Longitudinal monitoring of *Listeria monocytogenes* contamination patterns in a farmstead dairy processing facility. J Dairy Sci 90:2517–2524. doi:10.3168/jds.2006-392. 17430956

[5] Castro H, Jaakkonen A, Hakkinen M, Korkeala H, Lindström M. 2018. Occurrence, persistence, and contamination routes of *Listeria monocytogenes* genotypes on three Finnish dairy cattle farms: a longitudinal study. Appl Environ Microbiol 84:e02000-17. doi:10.1128/AEM.02000-17. 29222098 PMC5795088

[6] Samelis J, Metaxopoulos J. 1999. Incidence and principal sources of *Listeria* spp. and *Listeria monocytogenes* contamination in processed meats and a meat processing plant. Food Microbiol 16:465–477. doi:10.1006/fmic.1998.0263.

[7] Fox E, O’Mahony T, Clancy M, Dempsey R, O’Brien M, Jordan K. 2009. *Listeria monocytogenes* in the Irish dairy farm environment. J Food Prot 72:1450–1456. doi:10.4315/0362-028x-72.7.1450. 19681268

[8] Hellström S, Laukkanen R, Siekkinen KM, Ranta J, Maijala R, Korkeala H. 2010. *Listeria monocytogenes* contamination in pork can originate from farms. J Food Prot 73:641–648. doi:10.4315/0362-028X-73.4.641. 20377951

[9] Manson JM, Keis S, Smith JM, Cook GM. 2004. Acquired bacitracin resistance in *Enterococcus faecalis* is mediated by an ABC transporter and a novel regulatory protein, BcrR. Antimicrob Agents Chemother 48:3743–3748. doi:10.1128/AAC.48.10.3743-3748.2004. 15388429 PMC521867

[10] Aarnisalo K, Lundén J, Korkeala H, Wirtanen G. 2007. Susceptibility of *Listeria monocytogenes* strains to disinfectants and chlorinated alkaline cleaners at cold temperatures. LWT-Food Sci Technol 40:1041–1048. doi:10.1016/j.lwt.2006.07.009.

[11] Lundén J, Autio T, Markkula A, Hellström S, Korkeala H. 2003. Adaptive and cross-adaptive responses of persistent and non-persistent *Listeria monocytogenes* strains to disinfectants. Int J Food Microbiol 82:265–272. doi:10.1016/S0168-1605(02)00312-4. 12593929

[12] Keto-Timonen R, Tolvanen R, Lunden J, Korkeala H. 2007. An 8-year surveillance of the diversity and persistence of *Listeria monocytogenes* in a chilled food processing plant analyzed by amplified fragment length polymorphism. J Food Prot 70:1866–1873. doi:10.4315/0362-028x-70.8.1866. 17803143

[13] Stasiewicz MJ, Oliver HF, Wiedmann M, den Bakker HC. 2015. Whole-genome sequencing allows for improved identification of persistent *Listeria monocytogenes* in food-associated environments. Appl Environ Microbiol 81:6024–6037. doi:10.1128/AEM.01049-15. 26116683 PMC4551262

[14] Pasquali F, Palma F, Guillier L, Lucchi A, De Cesare A, Manfreda G. 2018. *Listeria monocytogenes* sequence types 121 and 14 repeatedly isolated within one year of sampling in a rabbit meat processing plant: persistence and ecophysiology. Front Microbiol 9:596. doi:10.3389/fmicb.2018.00596. 29662481 PMC5890179

[15] Hurley D, Luque-Sastre L, Parker CT, Huynh S, Eshwar AK, Nguyen SV, Andrews N, Moura A, Fox EM, Jordan K, Lehner A, Stephan R, Fanning S. 2019. Whole-genome sequencing-based characterization of 100 *Listeria monocytogenes* isolates collected from food processing environments over a four-year period. mSphere 4:e00252-19. doi:10.1128/mSphere.00252-19. 31391275 PMC6686224

[16] Harvey J, Gilmour A. 2001. Characterization of recurrent and sporadic *Listeria monocytogenes* isolates from raw milk and nondairy foods by pulsed-field gel electrophoresis, monocin typing, plasmid profiling, and cadmium and antibiotic resistance determination. Appl Environ Microbiol 67:840–847. doi:10.1128/AEM.67.2.840-847.2001. 11157252 PMC92656

[17] Pöntinen A, Aalto-Araneda M, Lindström M, Korkeala H. 2017. Heat resistance mediated by pLM58 plasmid-borne ClpL in *Listeria monocytogenes*. mSphere 2:e00364-17. doi:10.1128/mSphere.00364-17. 29104933 PMC5663981

[18] Naditz AL, Dzieciol M, Wagner M, Schmitz-Esser S. 2019. Plasmids contribute to food processing environment-associated stress survival in three *Listeria monocytogenes* ST121, ST8, and ST5 strains. Int J Food Microbiol 299:39–46. doi:10.1016/j.ijfoodmicro.2019.03.016. 30953994

[19] Hingston P, Brenner T, Truelstrup Hansen L, Wang S. 2019. Comparative analysis of *Listeria monocytogenes* plasmids and expression levels of plasmid-encoded genes during growth under salt and acid stress conditions. Toxins 11:426. doi:10.3390/toxins11070426. PMC6669625 31330827

[20] Müller A, Rychli K, Muhterem-Uyar M, Zaiser A, Stessl B, Guinane CM, Cotter PD, Wagner M, Schmitz-Esser S. 2013. Tn*6188*—a novel transposon in *Listeria monocytogenes* responsible for tolerance to benzalkonium chloride. PLoS One 8:e76835. doi:10.1371/journal.pone.0076835. 24098567 PMC3788773

[21] Meier-Kolthoff JP, Auch AF, Klenk HP, Göker M. 2013. Genome sequence-based species delimitation with confidence intervals and improved distance functions. BMC Bioinformatics 14:60–64. doi:10.1186/1471-2105-14-60. 23432962 PMC3665452

[22] Meier AB, Guldimann C, Markkula A, Pöntinen A, Korkeala H, Tasara T. 2017. Comparative phenotypic and genotypic analysis of Swiss and Finnish *Listeria monocytogenes* isolates with respect to benzalkonium chloride resistance. Front Microbiol 8:397. doi:10.3389/fmicb.2017.00397. 28386248 PMC5362634

[23] Garneau JE, Dupuis MÈ, Villion M, Romero DA, Barrangou R, Boyaval P, Fremaux C, Horvath P, Magadán AH, Moineau S. 2010. The CRISPR/Cas bacterial immune system cleaves bacteriophage and plasmid DNA. Nature 468:67–71. doi:10.1038/nature09523. 21048762

[24] Lee S, Ward TJ, Jima DD, Parsons C, Kathariou S. 2017. The arsenic resistance-associated *Listeria* genomic island LGI2 exhibits sequence and integration site diversity and a propensity for three *Listeria monocytogenes* clones with enhanced virulence. Appl Environ Microbiol 83:e01189-17. doi:10.1128/AEM.01189-17. 28842547 PMC5648914

[25] Pightling AW, Pettengill JB, Luo Y, Baugher JD, Rand H, Strain E. 2018. Interpreting whole-genome sequence analyses of foodborne bacteria for regulatory applications and outbreak investigations. Front Microbiol 9:1482. doi:10.3389/fmicb.2018.01482. 30042741 PMC6048267

[26] Maury MM, Bracq-Dieye H, Huang L, Vales G, Lavina M, Thouvenot P, Disson O, Leclercq A, Brisse S, Lecuit M. 2019. Hypervirulent *Listeria monocytogenes* clones’ adaption to mammalian gut accounts for their association with dairy products. Nat Commun 10:2488. doi:10.1038/s41467-019-10380-0. 31171794 PMC6554400

[27] Maury MM, Tsai Y-H, Charlier C, Touchon M, Chenal-Francisque V, Leclercq A, Criscuolo A, Gaultier C, Roussel S, Brisabois A, Disson O, Rocha EPC, Brisse S, Lecuit M. 2016. Uncovering *Listeria monocytogenes* hypervirulence by harnessing its biodiversity. Nat Genet 48:308–313. doi:10.1038/ng.3501. 26829754 PMC4768348

[28] Kuenne C, Voget S, Pischimarov J, Oehm S, Goesmann A, Daniel R, Hain T, Chakraborty T. 2010. Comparative analysis of plasmids in the genus *Listeria*. PLoS One 5:e12511. doi:10.1371/journal.pone.0012511. 20824078 PMC2932693

[29] Schmitz-Esser S, Anast JM, Cortes BW. 2021. A large-scale sequencing-based survey of plasmids in *Listeria monocytogenes* reveals global dissemination of plasmids. Front Microbiol 12:653155. doi:10.3389/fmicb.2021.653155. 33776982 PMC7994336

[30] Kuenne C, Billion A, Mraheil MA, Strittmatter A, Daniel R, Goesmann A, Barbuddhe S, Hain T, Chakraborty T. 2013. Reassessment of the *Listeria monocytogenes* pan-genome reveals dynamic integration hotspots and mobile genetic elements as major components of the accessory genome. BMC Genomics 14:47. doi:10.1186/1471-2164-14-47. 23339658 PMC3556495

[31] Ageorges V, Monteiro R, Leroy S, Burgess CM, Pizza M, Chaucheyras-Durand F, Desvaux M. 2020. Molecular determinants of surface colonisation in diarrhoeagenic *Escherichia coli* (DEC): from bacterial adhesion to biofilm formation. FEMS Microbiol Rev 44:314–350. doi:10.1093/femsre/fuaa008. 32239203

[32] Lee S, Ward TJ, Siletzky RM, Kathariou S. 2012. Two novel type II restriction-modification systems occupying genomically equivalent locations on the chromosomes of *Listeria monocytogenes* strains. Appl Environ Microbiol 78:2623–2630. doi:10.1128/AEM.07203-11. 22327591 PMC3318782

[33] León-Sampedro R, Novais C, Peixe L, Baquero F, Coque TM. 2016. Diversity and evolution of the Tn*5801*-*tet*(M)-like integrative and conjugative elements among *Enterococcus*. Antimicrob Agents Chemother 60:1736–1746. doi:10.1128/AAC.01864-15. 26729505 PMC4775984

[34] Tansirichaiya S, Rahman MA, Roberts AP. 2019. The transposon registry. Mobile DNA 10:1–6. doi:10.1186/s13100-019-0182-3. 31624505 PMC6785933

[35] Gilmour MW, Graham M, Van Domselaar G, Tyler S, Kent H, Trout-Yakel KM, Larios O, Allen V, Lee B, Nadon C. 2010. High-throughput genome sequencing of two *Listeria monocytogenes* clinical isolates during a large foodborne outbreak. BMC Genomics 11:120. doi:10.1186/1471-2164-11-120. 20167121 PMC2834635

[36] Palma F, Brauge T, Radomski N, Mallet L, Felten A, Mistou MY, Brisabois A, Guillier L, Midelet-Bourdin G. 2020. Dynamics of mobile genetic elements of *Listeria monocytogenes* persisting in ready-to-eat seafood processing plants in France. BMC Genomics 21:1–20. doi:10.1186/s12864-020-6544-x. PMC7006209 32028892

[37] Bertsch D, Uruty A, Anderegg J, Lacroix C, Perreten V, Meile L. 2013. Tn*6198*, a novel transposon containing the trimethoprim resistance gene *dfrG* embedded into a Tn*916* element in *Listeria monocytogenes*. J Antimicrob Chemother 68:986–991. doi:10.1093/jac/dks531. 23344576

[38] Lebrun M, Audurier A, Cossart P. 1994. Plasmid-borne cadmium resistance genes in *Listeria monocytogenes* are present on Tn*5422*, a novel transposon closely related to Tn*917*. J Bacteriol 176:3049–3061. doi:10.1128/jb.176.10.3049-3061.1994. 8188606 PMC205463

[39] Zink R, Loessner MJ, Scherer S. 1995. Charaterization of cryptic prophages (monocins) in *Listeria* and sequence analysis of a holin/endolysin gene. Microbiology 141:2577–2584. doi:10.1099/13500872-141-10-2577. 7582018

[40] Pasechnek A, Rabinovich L, Stadnyuk O, Azulay G, Mioduser J, Argov T, Borovok I, Sigal N, Herskovits AA. 2020. Active lysogeny in *Listeria monocytogenes* is a bacteria-phage adaptive response in the mammalian environment. Cell Rep 32:107956. doi:10.1016/j.celrep.2020.107956. 32726621 PMC7397523

[41] Price VJ, Huo W, Sharifi A, Palmer KL. 2016. CRISPR-Cas and restriction-modification act additively against conjugative antibiotic resistance plasmid transfer in *Enterococcus faecalis*. mSphere 1:e00064-16. doi:10.1128/mSphere.00064-16. 27303749 PMC4894674

[42] Bowran K, Palmer T. 2021. Extreme genetic diversity in the type VII secretion system of *Listeria monocytogenes* suggests a role in bacterial antagonism. Microbiology 167:e001034. doi:10.1099/mic.0.001034. PMC7613182 33599605

[43] Barrios AF, Zuo R, Ren D, Wood TK. 2006. Hha, YbaJ, and OmpA regulate *Escherichia coli* K12 biofilm formation and conjugation plasmids abolish motility. Biotechnol Bioeng 93:188–200. doi:10.1002/bit.20681. 16317765

[44] Parsons C, Lee S, Kathariou S. 2020. Dissemination and conservation of cadmium and arsenic resistance determinants in *Listeria* and other Gram‐positive bacteria. Mol Microbiol 113:560–569. doi:10.1111/mmi.14470. 31972871

[45] Smith AM, Tau NP, Smouse SL, Allam M, Ismail A, Ramalwa NR, Disenyeng B, Ngomane M, Thomas J. 2019. Outbreak of *Listeria monocytogenes* in South Africa, 2017–2018: laboratory activities and experiences associated with whole-genome sequencing analysis of isolates. Foodborne Pathog Dis 16:524–530. doi:10.1089/fpd.2018.2586. 31062992 PMC6653791

[46] Franz CM, Holzapfel WH, Stiles ME. 1999. Enterococci at the crossroads of food safety? Int J Food Microbiol 47:1–24. doi:10.1016/S0168-1605(99)00007-0. 10357269

[47] Jahan M, Holley RA. 2016. Transfer of antibiotic resistance from *Enterococcus faecium* of fermented meat origin to *Listeria monocytogenes* and *Listeria innocua*. Lett Appl Microbiol 62:304–310. doi:10.1111/lam.12553. 26854329

[48] Haubert L, da Cunha CE, Lopes GV, da Silva WP. 2018. Food isolate *Listeria monocytogenes* harboring *tetM* gene plasmid-mediated exchangeable to *Enterococcus faecalis* on the surface of processed cheese. Food Res Int 107:503–508. doi:10.1016/j.foodres.2018.02.062. 29580513

[49] Aarestrup FM, Kruse H, Tast E, Hammerum AM, Jensen LB. 2000. Associations between the use of antimicrobial agents for growth promotion and the occurrence of resistance among *Enterococcus faecium* from broilers and pigs in Denmark, Finland, and Norway. Microb Drug Resist 6:63–70. doi:10.1089/mdr.2000.6.63. 10868809

[50] Chen MY, Lira F, Liang HQ, Wu RT, Duan JH, Liao XP, Martínez JL, Liu YH, Sun J. 2016. Multilevel selection of *bcrABDR*-mediated bacitracin resistance in *Enterococcus faecalis* from chicken farms. Sci Rep 6:34895–34897. doi:10.1038/srep34895. 27731342 PMC5059624

[51] Harrison E, Brockhurst MA. 2017. Ecological and evolutionary benefits of temperate phage: what does or doesn't kill you makes you stronger. BioEssays 39:1700112. doi:10.1002/bies.201700112. 28983932

[52] Pitcher DG, Saunders NA, Owen RJ. 1989. Rapid extraction of bacterial genomic DNA with guanidium thiocyanate. Lett Appl Microbiol 8:151–156. doi:10.1111/j.1472-765X.1989.tb00262.x.

[53] Bolger AM, Lohse M, Usadel B. 2014. Trimmomatic: a flexible trimmer for Illumina sequence data. Bioinformatics 30:2114–2120. doi:10.1093/bioinformatics/btu170. 24695404 PMC4103590

[54] Bankevich A, Nurk S, Antipov D, Gurevich AA, Dvorkin M, Kulikov AS, Lesin VM, Nikolenko SI, Pham S, Prjibelski AD, Pyshkin AV, Sirotkin AV, Vyahhi N, Tesler G, Alekseyev MA, Pevzner PA. 2012. SPAdes: a new genome assembly algorithm and its applications to single-cell sequencing. J Comput Biol 19:455–477. doi:10.1089/cmb.2012.0021. 22506599 PMC3342519

[55] Gurevich A, Saveliev V, Vyahhi N, Tesler G. 2013. QUAST: quality assessment tool for genome assemblies. Bioinformatics 29:1072–1075. doi:10.1093/bioinformatics/btt086. 23422339 PMC3624806

[56] Wood DE, Salzberg SL. 2014. Kraken: ultrafast metagenomic sequence classification using exact alignments. Genome Biol 15:R46–2. doi:10.1186/gb-2014-15-3-r46. 24580807 PMC4053813

[57] Seemann T. 2014. Prokka: rapid prokaryotic genome annotation. Bioinformatics 30:2068–2069. doi:10.1093/bioinformatics/btu153. 24642063

[58] Page AJ, Cummins CA, Hunt M, Wong VK, Reuter S, Holden MT, Fookes M, Falush D, Keane JA, Parkhill J. 2015. Roary: rapid large-scale prokaryote pan genome analysis. Bioinformatics 31:3691–3693. doi:10.1093/bioinformatics/btv421. 26198102 PMC4817141

[59] Ragon M, Wirth T, Hollandt F, Lavenir R, Lecuit M, Le Monnier A, Brisse S. 2008. A new perspective on *Listeria monocytogenes* evolution. PLoS Pathog 4:e1000146. doi:10.1371/journal.ppat.1000146. 18773117 PMC2518857

[60] Moura A, Criscuolo A, Pouseele H, Maury MM, Leclercq A, Tarr C, Björkman JT, Dallman T, Reimer A, Enouf V, Larsonneur E, Carleton H, Bracq-Dieye H, Katz LS, Jones L, Touchon M, Tourdjman M, Walker M, Stroika S, Cantinelli T, Chenal-Francisque V, Kucerova Z, Rocha EPC, Nadon C, Grant K, Nielsen EM, Pot B, Gerner-Smidt P, Lecuit M, Brisse S. 2016. Whole genome-based population biology and epidemiological surveillance of *Listeria monocytogenes*. Nat Microbiol 2:16185. doi:10.1038/nmicrobiol.2016.185. 27723724 PMC8903085

[61] Katz LS, Griswold T, Williams-Newkirk AJ, Wagner D, Petkau A, Sieffert C, Van Domselaar G, Deng X, Carleton HA. 2017. A comparative analysis of the Lyve-SET phylogenomics pipeline for genomic epidemiology of foodborne pathogens. Front Microbiol 8:375. doi:10.3389/fmicb.2017.00375. 28348549 PMC5346554

[62] Croucher NJ, Page AJ, Connor TR, Delaney AJ, Keane JA, Bentley SD, Parkhill J, Harris SR. 2015. Rapid phylogenetic analysis of large samples of recombinant bacterial whole genome sequences using Gubbins. Nucleic Acids Res 43:e15. doi:10.1093/nar/gku1196. 25414349 PMC4330336

[63] Guindon S, Dufayard JF, Lefort V, Anisimova M, Hordijk W, Gascuel O. 2010. New algorithms and methods to estimate maximum-likelihood phylogenies: assessing the performance of PhyML 3.0. Syst Biol 59:307–321. doi:10.1093/sysbio/syq010. 20525638

[64] Wick RR, Schultz MB, Zobel J, Holt KE. 2015. Bandage: interactive visualization of *de novo* genome assemblies. Bioinformatics 31:3350–3352. doi:10.1093/bioinformatics/btv383. 26099265 PMC4595904

[65] Kumar S, Stecher G, Tamura K. 2016. MEGA7: molecular evolutionary genetics analysis version 7.0 for bigger datasets. Mol Biol Evol 33:1870–1874. doi:10.1093/molbev/msw054. 27004904 PMC8210823

[66] Alikhan NF, Petty NK, Zakour NL, Beatson SA. 2011. BLAST Ring Image Generator (BRIG): simple prokaryote genome comparisons. BMC Genomics 12:1–10. doi:10.1186/1471-2164-12-402. PMC3163573 21824423

[67] Sullivan MJ, Petty NK, Beatson SA. 2011. Easyfig: a genome comparison visualizer. Bioinformatics 27:1009–1010. doi:10.1093/bioinformatics/btr039. 21278367 PMC3065679

[68] Arndt D, Grant JR, Marcu A, Sajed T, Pon A, Liang Y, Wishart DS. 2016. PHASTER: a better, faster version of the PHAST phage search tool. Nucleic Acids Res 44:W16–W21. doi:10.1093/nar/gkw387. 27141966 PMC4987931

[69] Meier-Kolthoff JP, Göker M. 2017. VICTOR: genome-based phylogeny and classification of prokaryotic viruses. Bioinformatics 33:3396–3404. doi:10.1093/bioinformatics/btx440. 29036289 PMC5860169

[70] Miettinen MK, Björkroth KJ, Korkeala HJ. 1999. Characterization of *Listeria monocytogenes* from an ice cream plant by serotyping and pulsed-field gel electrophoresis. Int J Food Microbiol 46:187–192. doi:10.1016/s0168-1605(98)00185-8. 10100898

[71] Göker M, García-Blázquez G, Voglmayr H, Tellería MT, Martín MP. 2009. Molecular taxonomy of phytopathogenic fungi: a case study in *Peronospora*. PLoS One 4:e6319. doi:10.1371/journal.pone.0006319. 19641601 PMC2712678

[72] Brynildsrud O, Bohlin J, Scheffer L, Eldholm V. 2016. Rapid scoring of genes in microbial pan-genome-wide association studies with Scoary. Genome Biol 17:1–9. doi:10.1186/s13059-016-1132-8. 27887642 PMC5124306

