# Erratum for Castro et al., “Mobile Elements Harboring Heavy Metal and Bacitracin Resistance Genes Are Common among Listeria monocytogenes Strains Persisting on Dairy Farms”

**DOI:** 10.1128/mSphere.00733-21

**Published:** 2021-09-15

**Authors:** Hanna Castro, François P. Douillard, Hannu Korkeala, Miia Lindström

**Affiliations:** a Department of Food Hygiene and Environmental Health, Faculty of Veterinary Medicine, University of Helsinki, Helsinki, Finland

## ERRATUM

Volume 6, no. 4, e00383-21, 2021, https://doi.org/10.1128/mSphere.00383-21.

Page 5: Table 1 in the published article contains errors in the *N*, Mean, and Maximum columns. The corrected table is shown here.

**TABLE 1:** Pairwise distances within persistent clusters of L. monocytogenes from dairy farms A to C

Cluster	CC^*a*^	ST^*b*^	CT^*c*^	*N* ^*d*^	Farm(s)	Pairwise distance (no. of SNPs)		
						Mean	Minimum	Maximum
C1	8	8	9176	8	A	1.5	0	4
C2	14	14	9177	34	A	2.1	0	8
C3	14	91	9178	8	A	2.0	0	6
C4	14	91	9179	21	A, B	2.5	0	8
C5	18	18	9180	9	B	3.0	0	8
C6	18	18	9181	6	B	1.7	0	10
C7	20	20	9182	32	C	2.4	0	7
C8	20	20	9189	5	B	3.2	0	8
C9	20	20	9183	7	A	4.3	0	9
C10	20	20	9184	5	B	2.4	0	6
C11	20	20	9185	9	C	5.4	0	10
C12	20	20	9186	4	B	1.5	0	3
C13	37	37	9187	20	A	2.4	0	6
C14	37	37	9188, 9205	9	A	6.4	0	16
All clusters						2.9	0	8

a CC, clonal complex.

b ST, multilocus sequence typing (MLST) profile.

c CT, core genome multilocus sequence typing (cgMLST) profile.

d No. of isolates in the persistent cluster.

Page 11, line 10: “In contrast, genes associated with the CRISPR-*cas* type IIA system and the type II restriction-modification system LmoJ3 (24) were negatively associated with nonpersistence” should read “In contrast, genes associated with the CRISPR-*cas* type IIA system and the type II restriction-modification system LmoJ3 (24) were associated with nonpersistence.”

Page 15: References 24 and 32 are interchanged. The correctly numbered references are as follows:

24. Lee S, Ward TJ, Siletzky RM, Kathariou S. 2012. Two novel type II restriction-modification systems occupying genomically equivalent locations on the chromosomes of *Listeria monocytogenes strains*. Appl Environ Microbiol 78:2623–2630. https://doi.org/10.1128/AEM.07203-11.

32. Lee S, Ward TJ, Jima DD, Parsons C, Kathariou S. 2017. The arsenic resistance-associated Listeria genomic island LGI2 exhibits sequence and integration site diversity and a propensity for three *Listeria monocytogenes* clones with enhanced virulence. Appl Environ Microbiol 83:e01189-17. https://doi.org/10.1128/AEM.01189-17.

