# Supplementary figures and images for: Mobile Elements Harboring Heavy Metal and Bacitracin Resistance Genes Are Common among Listeria monocytogenes Strains Persisting on Dairy Farms
Source: mSphere. 2021 Jul 7;6(4):e00383-21. doi: 10.1128/mSphere.00383-21 (PMC8386393; doi:10.1128/mSphere.00383-21)

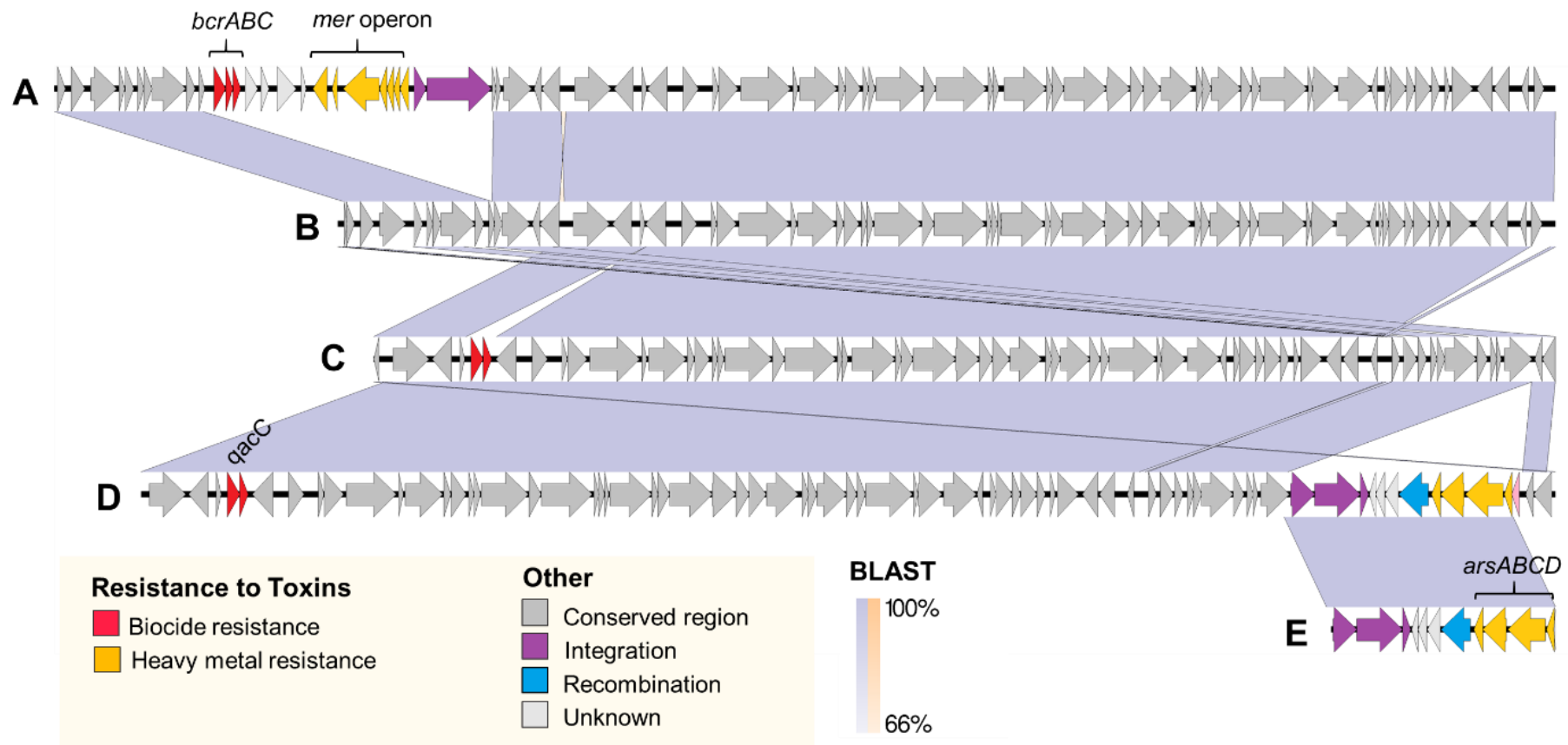

Supplement: FIG S1 [file msphere.00383-21-sf001.pdf]

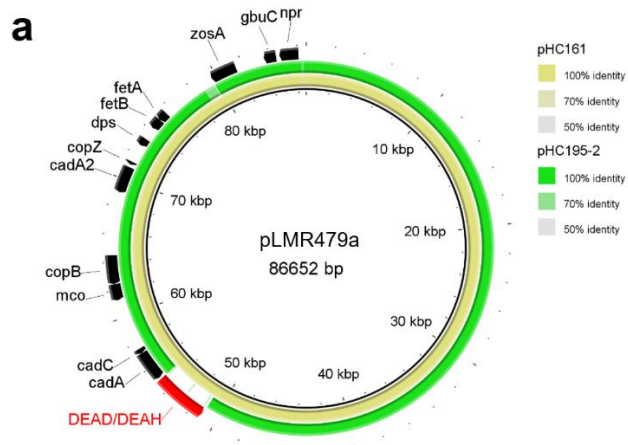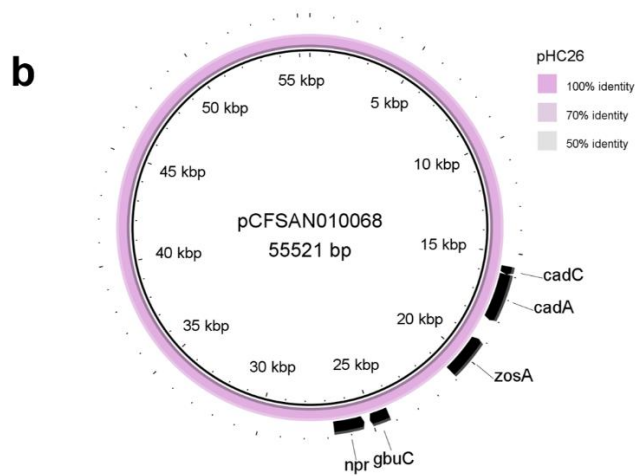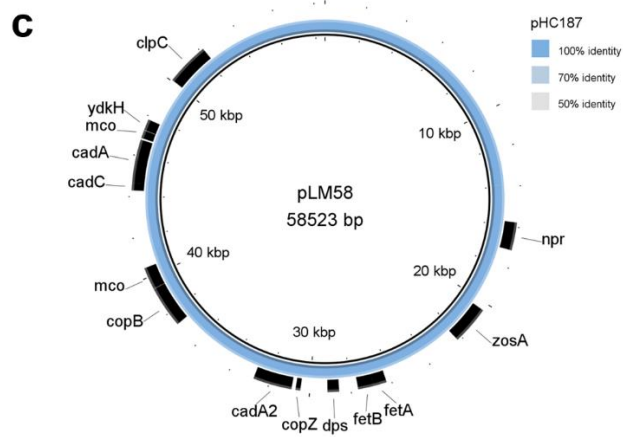

Supplement: FIG S2 [file msphere.00383-21-sf002.pdf]

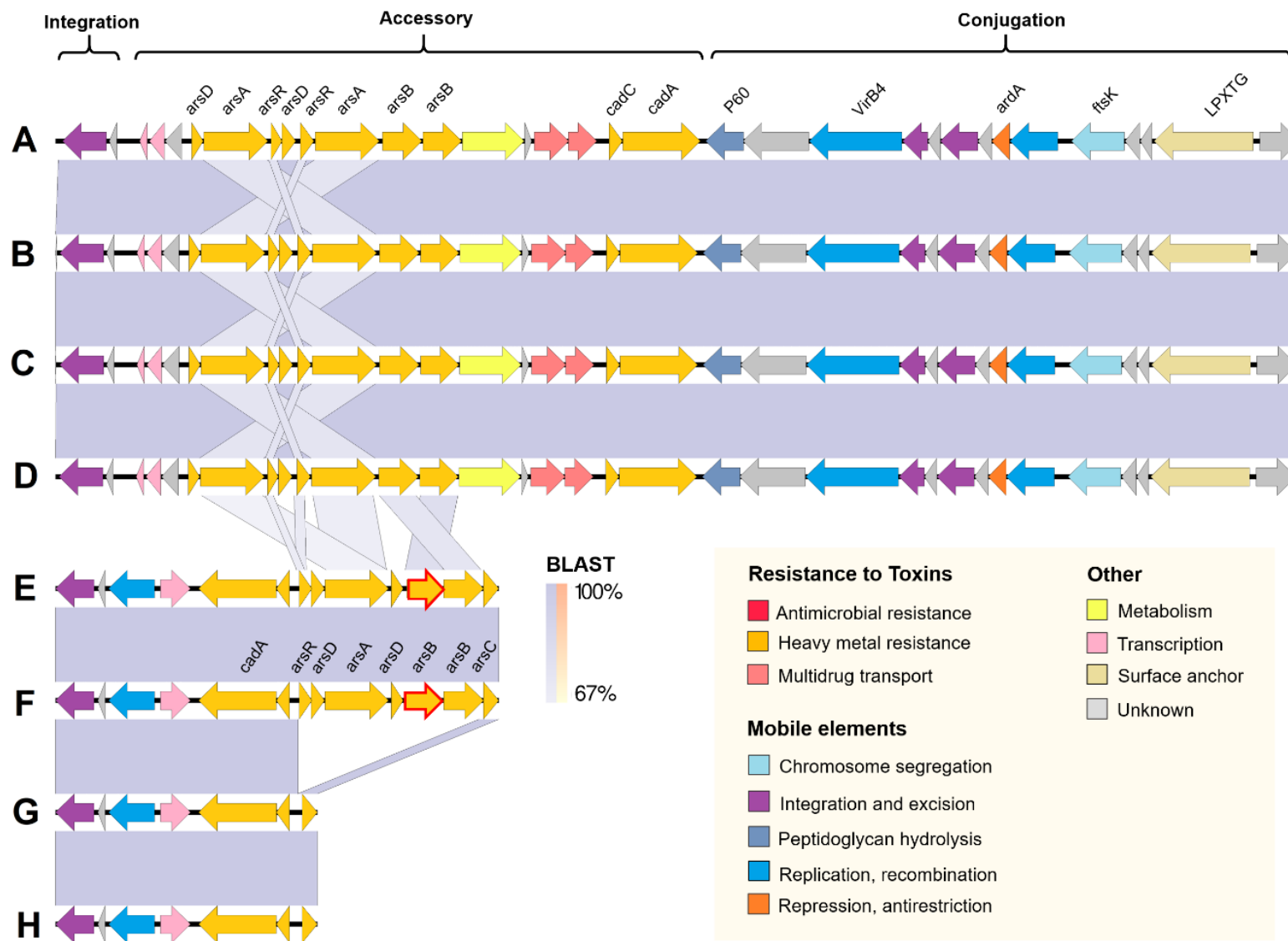

Supplement: FIG S3 [file msphere.00383-21-sf003.pdf]

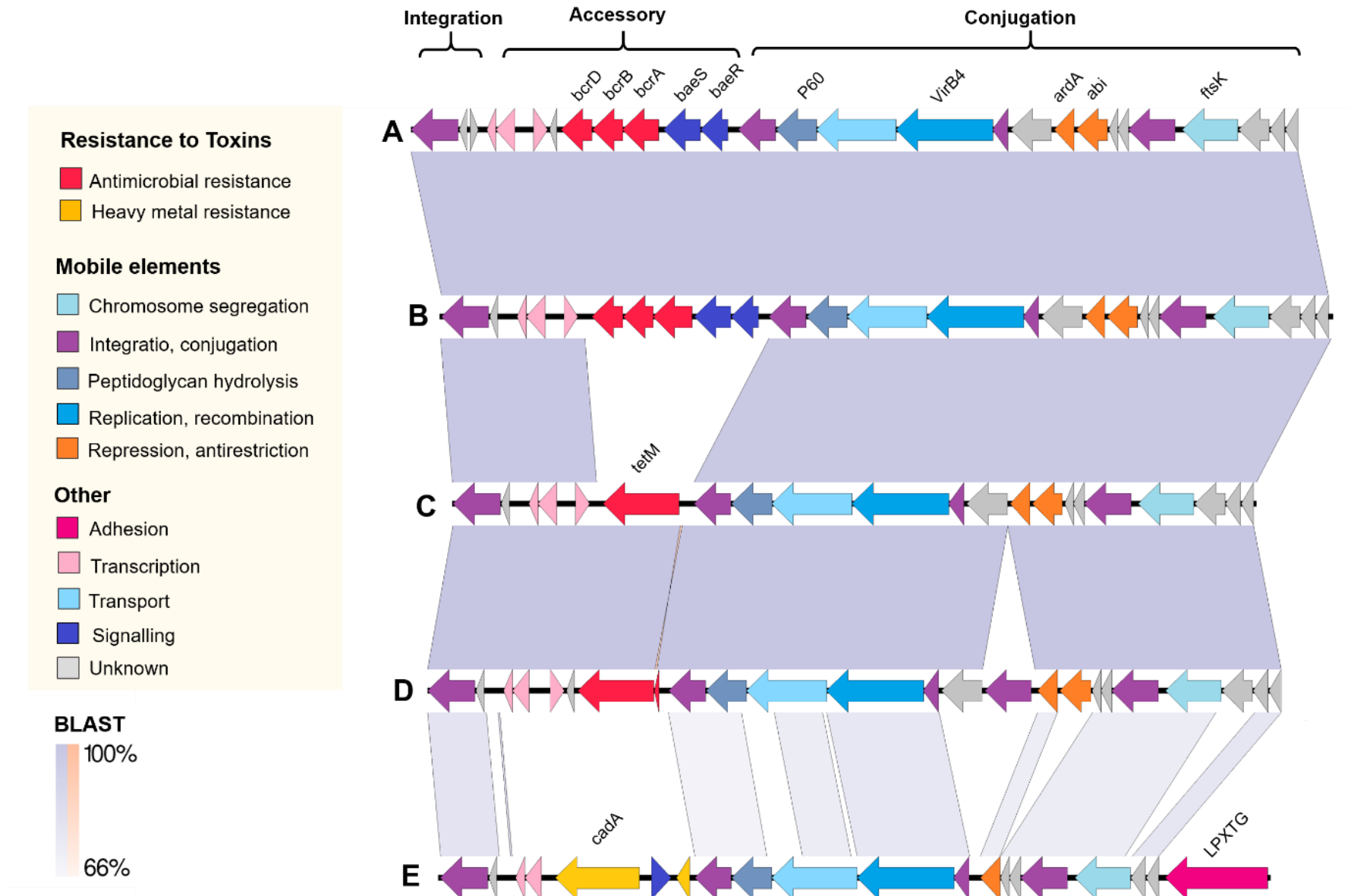

Supplement: FIG S4 [file msphere.00383-21-sf004.pdf]
