# Supplementary material for: Mobile Elements Harboring Heavy Metal and Bacitracin Resistance Genes Are Common among Listeria monocytogenes Strains Persisting on Dairy Farms
Source: mSphere. 2021 Jul 7;6(4):e00383-21. doi: 10.1128/mSphere.00383-21 (PMC8386393; doi:10.1128/mSphere.00383-21)
Supplement: FIG S5 [file msphere.00383-21-sf005.pdf]

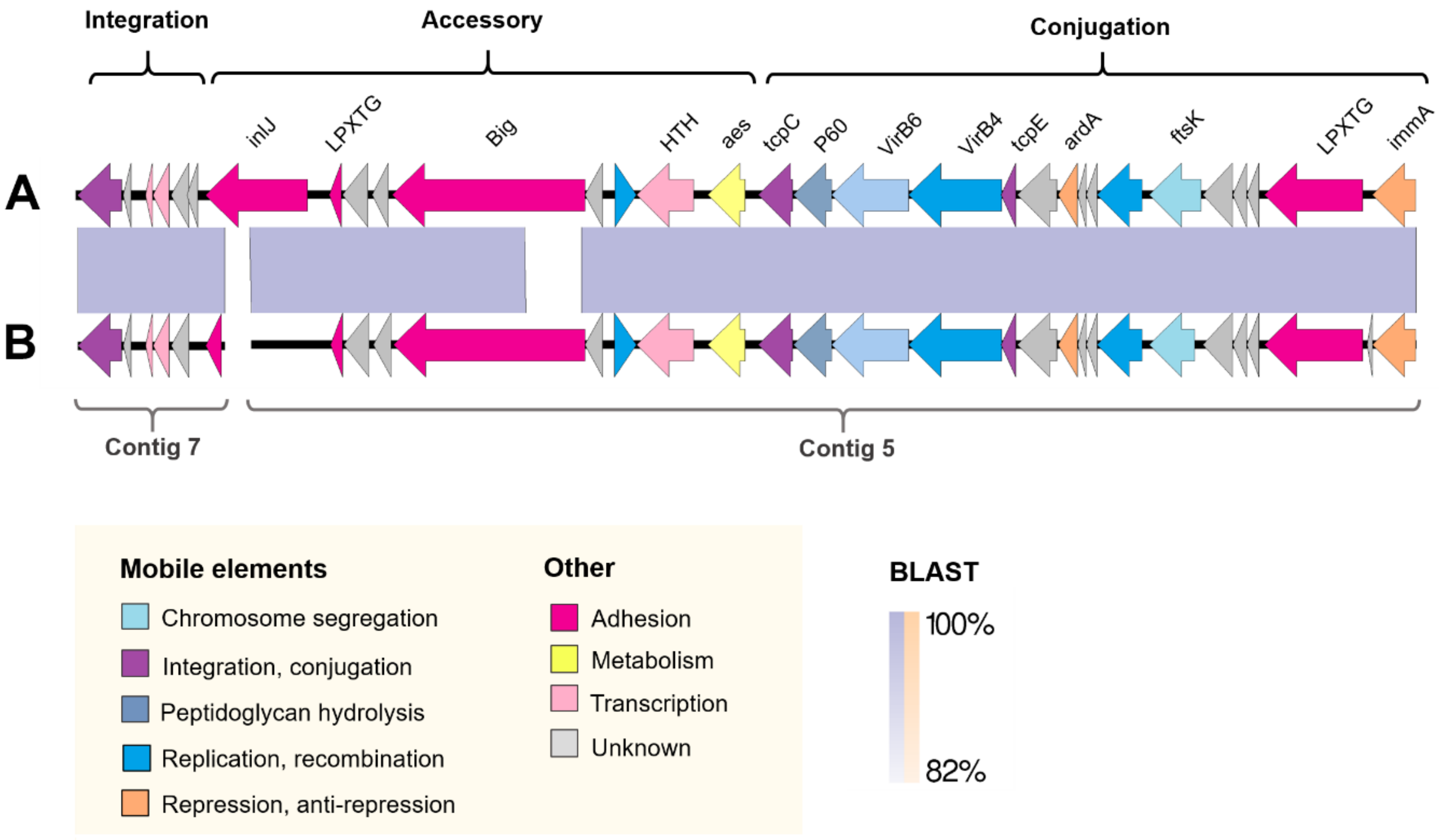
Integration
Accessory
Conjugation
inlJ
LPXTG
Big
HTH
aes
tcpC
P60
VirB6
VirB4
tcpE
ardA
ftsK
LPXTG
immA
A
B
Contig 7
Contig 5
Mobile elements
Chromosome segregation
Integration, conjugation
Peptidoglycan hydrolysis
Replication, recombination
Repression, anti-repression
Other
Adhesion
Metabolism
Transcription
Unknown
BLAST
100%
82%
