# Supplementary material for: Mobile Elements Harboring Heavy Metal and Bacitracin Resistance Genes Are Common among Listeria monocytogenes Strains Persisting on Dairy Farms
Source: mSphere. 2021 Jul 7;6(4):e00383-21. doi: 10.1128/mSphere.00383-21 (PMC8386393; doi:10.1128/mSphere.00383-21)
Supplement: FIG S6 [file msphere.00383-21-sf006.pdf]

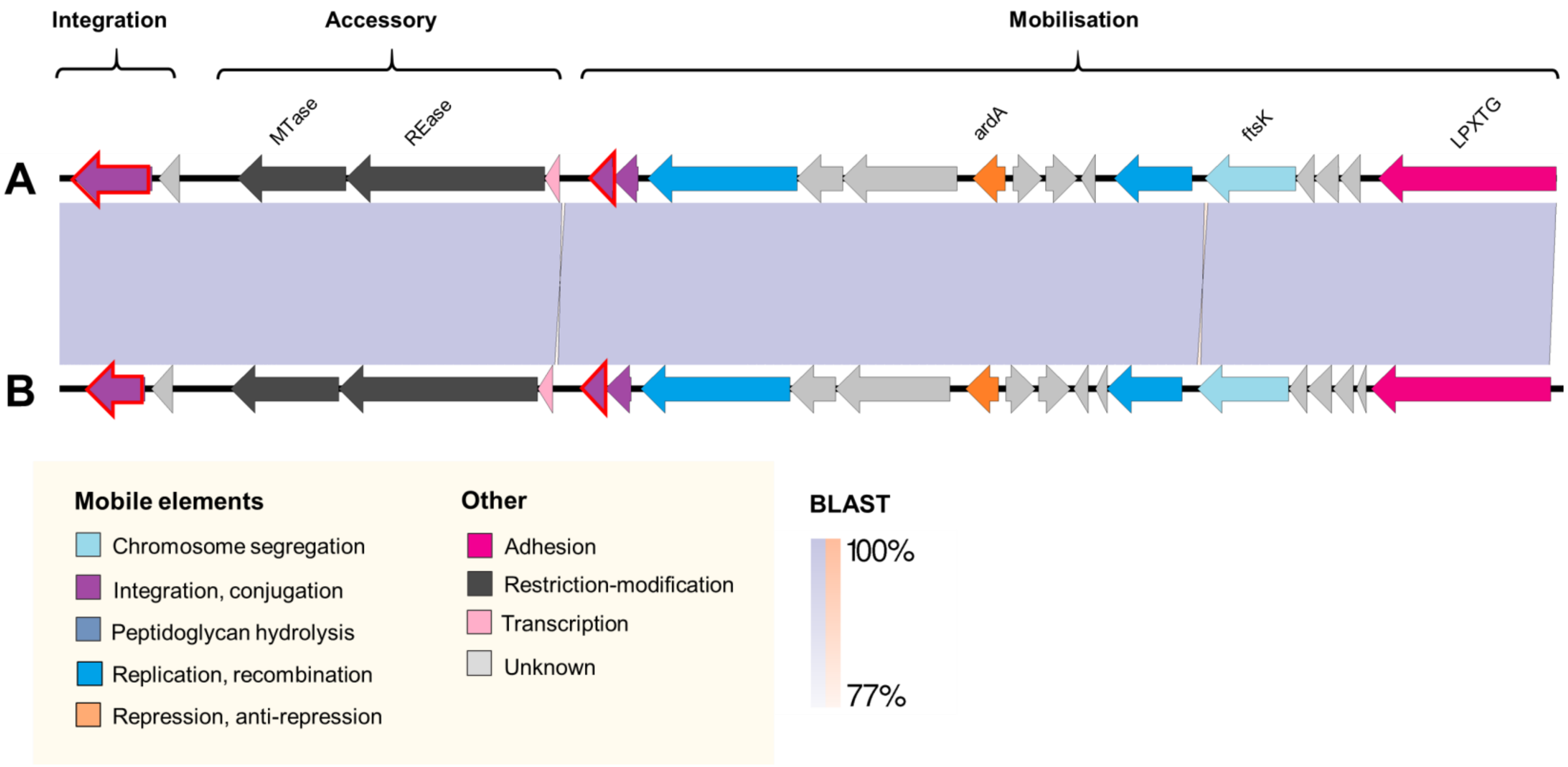

Integration
Accessory
Mobilisation
MTase
REase
ardA
ftsK
LPXTG
A
B
Mobile elements
Chromosome segregation
Integration, conjugation
Peptidoglycan hydrolysis
Replication, recombination
Repression, anti-repression
Other
Adhesion
Restriction-modification
Transcription
Unknown
BLAST
100%
77%
