# Supplementary material for: Mobile Elements Harboring Heavy Metal and Bacitracin Resistance Genes Are Common among Listeria monocytogenes Strains Persisting on Dairy Farms
Source: mSphere. 2021 Jul 7;6(4):e00383-21. doi: 10.1128/mSphere.00383-21 (PMC8386393; doi:10.1128/mSphere.00383-21)
Supplement: FIG S7 [file msphere.00383-21-sf007.pdf]

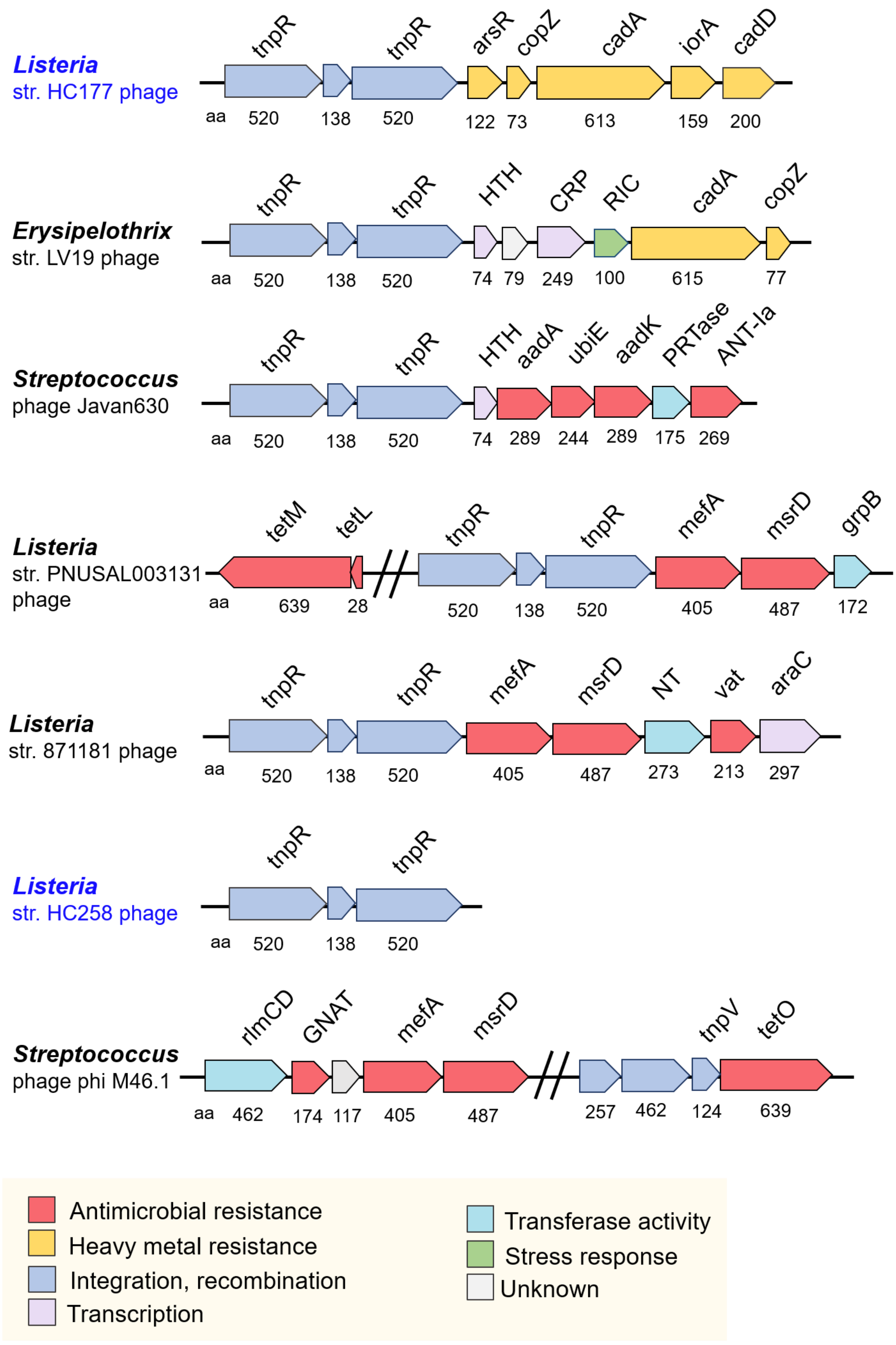

Listeria
str. HC177 phage
tnpR
tnpR
arsR
copZ
cadA
iorA
cadD
aa 520 138 520 122 73 613 159 200
Erysipelothrix
str. LV19 phage
tnpR
tnpR
HTH
CRP
RIC
cadA
copZ
aa 520 138 520 74 79 249 100 615 77
Streptococcus
phage Javan630
tnpR
tnpR
HTH
aadA
ubiE
aadK
PRTase
ANT-Ia
aa 520 138 520 74 289 244 289 175 269
Listeria
str. PNUSAL003131
phage
tetM
tetL
tnpR
tnpR
mefA
msrD
grpB
aa 639 28 520 138 520 405 487 172
Listeria
str. 871181 phage
tnpR
tnpR
mefA
msrD
NT
vat
araC
aa 520 138 520 405 487 273 213 297
Listeria
str. HC258 phage
tnpR
tnpR
aa 520 138 520
Streptococcus
phage phi M46.1
rlmCD
GNAT
mefA
msrD
tnpV
tetO
aa 462 174 117 405 487 257 462 124 639
Antimicrobial resistance
Heavy metal resistance
Integration, recombination
Transcription
Transferase activity
Stress response
Unknown
