# Supplementary material for: Mobile Elements Harboring Heavy Metal and Bacitracin Resistance Genes Are Common among Listeria monocytogenes Strains Persisting on Dairy Farms
Source: mSphere. 2021 Jul 7;6(4):e00383-21. doi: 10.1128/mSphere.00383-21 (PMC8386393; doi:10.1128/mSphere.00383-21)
Supplement: TABLE S2 [file msphere.00383-21-st002.pdf]

Table S2. Genes positively or negatively associated (Bonferroni-corrected *p*<.05) with persistent clades among the 233 *L. monocytogenes* Lineage II isolates of this study.

Element	Protein Accession	Annotation or domain	Odds ratio	Bonferroni p
type VII secretion system	WP_026750033	ESAT-6 secretion machinery protein EssC	7.11	1.5E-04
type VII secretion system	WP_026750032	DUF5082	7.11	1.5E-04
type VII secretion system	WP_003731422	hypothetical protein	6.95	1.9E-04
hypervariable hotspot 4	WP_061668643	hypothetical protein	inf	2.1E-07
hypervariable hotspot 8	WP_003733794	hypothetical protein	4.60	5.8E-03
hypervariable hotspot 8	WP_026749868	immunity protein (Imm-NTF2 -domain)	4.60	5.8E-03
hypervariable hotspot 8	WP_003721523	DUF4274	4.49	1.1E-02
IS3-like mobile element	EAG2033583	SAM-dependent methyltransferase, partial	6.14	8.3E-05
phage	YP_009907771	hypothetical protein	8.33	6.7E-07
phage	YP_009044813	hypothetical protein	6.23	4.3E-05
	WP_003722049	1,4-beta-N-acetylmuramoylhydrolase	10.01	4.6E-04
	WP_003722051	ribonuclease BN	7.21	4.3E-06
	WP_009933017	hypothetical protein	7.73	2.1E-02
	WP_026749741	biofilm-associated protein BapL	7.11	1.5E-04
	WP_010989981	hypothetical protein	4.33	4.9E-02
CRISPR-cas IIA	WP_014601172	CRISPR-associated endonuclease Cas9	0.00	3.2E-03
CRISPR-cas IIA	WP_014601170	CRISPR-associated protein Csn2	0.00	6.7E-04
CRISPR-cas IIA	WP_003723648	CRISPR-associated endoribonuclease Cas2	0.00	6.7E-04
CRISPR-cas IIA	WP_009925355	CRISPR-associated protein Cas4	0.00	2.8E-14
CRISPR-cas IIA	WP_014601171	CRISPR-associated endonuclease Cas1	0.00	6.7E-04
type VII secretion system	WP_05431447	ESAT-6 secretion machinery protein	0.09	4.9E-08
type VII secretion system	WP_009924118	DUF4176	0.09	4.9E-08
type VII secretion system	WP_003724890	hypothetical protein	0.09	4.9E-08
type VII secretion system	WP_009924116	hypothetical protein	0.09	4.9E-08
type VII secretion system	WP_070275768	hypothetical protein	0.00	3.9E-08
type VII secretion system	WP_031674920	toxin B	0.00	3.9E-08
hypervariable hotspot 8	WP_012951470	hypothetical protein	0.22	5.8E-03
hypervariable hotspot 8	WP_009931454	hypothetical protein	0.21	5.0E-03
hypervariable hotspot 8	WP_014931350	DUF3130	0.17	1.6E-04
hypervariable hotspot 8	ADB67935	VOC family protein	0.05	6.7E-10
hypervariable hotspot 8	WP_052672896	chromosome segregation ATPase	0.00	2.8E-05
hypervariable hotspot 8	WP_045131475	hypothetical protein	0.00	2.8E-05
hypervariable hotspot 8	WP_031664941	recombination and strand exchange inhibitor	0.00	5.6E-06
hypervariable hotspot 8	WP_045131476	hypothetical protein	0.00	2.8E-05
hypervariable hotspot 8	WP_014600726	AraC family transcriptional regulator	0.00	4.0E-11
LmoJ3	WP_061661643	DNA cytosine methyltransferase	0.00	2.8E-05
LmoJ3	WP_069000933	NgoFVII family restriction endonuclease	0.00	2.8E-05
LmoJ3	WP_069000932	XRE-family transcriptional regulator	0.00	2.8E-05
phage	YP_009907759	hypothetical protein	0.13	2.1E-02
phage	YP_009044855	hypothetical protein	0.07	2.7E-02
phage	WP_003725093	hypothetical protein	0.05	2.9E-02
phage	WP_003725092	KTSC domain	0.05	2.9E-02

phage	WP_009917698	hypothetical protein	0.04	2.1E-07
phage	NP_463476	phage_Gp15 domain	0.03	6.0E-03
phage	WP_014930203	DUF1642	0.02	1.4E-03
phage	YP_001468391	phage_GP20	0.02	3.0E-04
phage	NP_463533	gp68	0.02	1.3E-05
phage	WP_020830757	hypothetical protein	0.00	6.7E-04
phage	NP_463467	major capsid protein	0.00	2.8E-05
phage	WP_003731437	XRE family transcriptional regulator	0.00	1.5E-02
phage	WP_014601102	XRE family transcriptional regulator	0.00	1.5E-02
phage	YP_001468393	gp7	0.00	6.7E-04
phage	YP_009907764	YozE_SAM_like -domain	0.00	2.8E-05
phage	YP_009907767	hypothetical protein	0.00	1.5E-02
phage	NP_463462	putative terminase small subunit	0.00	2.8E-05
phage	YP_009907763	XRE-family transcriptional regulator	0.00	3.2E-03
phage	WP_097528476	hypothetical protein	0.00	3.2E-03
phage	YP_008126752	Sipho_Gp157	0.00	3.2E-03
phage	YP_009907755	GP47 protein	0.00	1.5E-02
phage	WP_003733717	helix-turn-helix domain-containing protein	0.00	1.5E-02
	WP_054314211	cell wall anchor protein	0.10	9.6E-07
