# Supplementary material for: Mobile Elements Harboring Heavy Metal and Bacitracin Resistance Genes Are Common among Listeria monocytogenes Strains Persisting on Dairy Farms
Source: mSphere. 2021 Jul 7;6(4):e00383-21. doi: 10.1128/mSphere.00383-21 (PMC8386393; doi:10.1128/mSphere.00383-21)
Supplement: TABLE S1 [file msphere.00383-21-st001.pdf]

Table S1. Phage genomes obtained from GenBank that were used to aid the phylogenetic and taxonomic classification of prophages detected among the 250 *Listeria monocytogenes* genomes from this study.

Phage name	Accession	Family	Genus	Host
A006	NC_009815.1	*Siphoviridae*	unclassified	*Listeria*
A118	NC_003216.1	*Siphoviridae*	unclassified	*Listeria*
A500	NC_009810.1	*Siphoviridae*	unclassified	*Listeria*
A511	NC_009811.2	*Herelleviridae*	*Pecentumvirus*	*Listeria*
B025	NC_009812.1	*Siphoviridae*	*Psavirus*	*Listeria*
B054	NC_009813.1	*Myoviridae*	unclassified	*Listeria*
Javan630	MK448997.1	*Siphoviridae*	unclassified	*Streptococcus*
LP-030-2	NC_021539.2	*Siphoviridae*	*Psavirus*	*Listeria*
LP-030-3	NC_024384.1	*Siphoviridae*	unclassified	*Listeria*
LP-037	NC_021787.2	*Siphoviridae*	*Homburgvirus*	*Listeria*
LP-048	NC_024359.1	*Herelleviridae*	*Pecentumvirus*	*Listeria*
LP-101	NC_024387.1	*Siphoviridae*	*Psavirus*	*Listeria*
LP-HM00113468	MT500540.1	*Siphoviridae*	*Psavirus*	*Listeria*
P100	NC_007610.1	*Herelleviridae*	*Pecentumvirus*	*Listeria*
P70	NC_018831.1	*Siphoviridae*	*Homburgvirus*	*Listeria*
PSA	NC_003291	*Siphoviridae*	*Psavirus*	*Listeria*
PSU-VKH-LP019	MH341451	*Siphoviridae*	unclassified	*Listeria*
PSU-VKH-LP040	MH341452	*Siphoviridae*	unclassified	*Listeria*
PSU-VKH-LP041	MH341453	*Myoviridae*	unclassified	*Listeria*
vB_LmoS_188	NC_028871.1	*Siphoviridae*	unclassified	*Listeria*
